# Clinical interventions that influence vaginal birth after cesarean delivery rates: Systematic Review & Meta-Analysis

**DOI:** 10.1186/s12884-019-2689-5

**Published:** 2019-12-30

**Authors:** Aireen Wingert, Lisa Hartling, Meghan Sebastianski, Cydney Johnson, Robin Featherstone, Ben Vandermeer, R. Douglas Wilson

**Affiliations:** 1 Department of Pediatrics, Alberta Research Centre for Health Evidence, University of Alberta, Edmonton, Alberta Canada; 2 Alberta Strategy for Patient-Oriented Research (SPOR) SUPPORT Unit Knowledge Translation Platform, University of Alberta, Edmonton, Alberta Canada; 3 Department of Obstetrics and Gynecology, Cumming School of Medicine, University of Calgary, 1403 – 29 Street NW, Calgary, AB T2N 2T9 Canada

**Keywords:** Vaginal birth after cesarean, Trial of labor after cesarean, Systematic review, Meta-analysis

## Abstract

**Background:**

To systematically review the literature on clinical interventions that influence vaginal birth after cesarean (VBAC) rates.

**Methods:**

We searched Ovid Medline, Ovid Embase, Wiley Cochrane Library, CINAHL via EBSCOhost; and Ovid PsycINFO. Additional studies were identified by searching for clinical trial records, conference proceedings and dissertations. Limits were applied for language (English and French) and year of publication (1985 to present). Two reviewers independently screened comparative studies (randomized or non-randomized controlled trials, and observational designs) according to a priori eligibility criteria: women with prior cesarean sections; any clinical intervention or exposure intended to increase the VBAC rate; any comparator; and, outcomes reporting VBAC, uterine rupture and uterine dehiscence rates. One reviewer extracted data and a second reviewer verified for accuracy. Meta-analysis was conducted using Mantel-Haenszel (random effects model) relative risks (VBAC rate) and risk differences (uterine rupture and dehiscence). Two reviewers independently conducted methodological quality assessments using the Mixed Methods Appraisal Tool (MMAT).

**Results:**

Twenty-nine studies (six trials and 23 cohorts) examined different clinical interventions affecting rates of vaginal deliveries among women with a prior cesarean delivery (CD). Methodological quality was good overall for the trials; however, concerns among the cohort studies regarding selection bias, comparability of groups and outcome measurement resulted in higher risk of bias. Interventions for labor induction, with or without cervical ripening, included pharmacologic (oxytocin, prostaglandins, misoprostol, mifepristone, epidural analgesia), non-pharmacologic (membrane sweep, amniotomy, balloon devices), and combined (pharmacologic and non-pharmacologic). Single studies with small sample sizes and event rates contributed to most comparisons, with no clear differences between groups on rates of VBAC, uterine rupture and uterine dehiscence.

**Conclusions:**

This systematic review evaluated clinical interventions directed at increasing the rate of vaginal delivery among women with a prior CD and found low to very low certainty in the body of evidence for cervical ripening and/or labor induction techniques. There is insufficient high-quality evidence to inform optimal clinical interventions among women attempting a trial of labor after a prior CD.

## Background

Over 103,000 cesarean deliveries (CDs) occurred in 2017 within Canadian hospitals [1]. In Canada, CDs continue to be the leading inpatient surgery with elective/scheduled CD as a main contributor. Since 1997, the rate of CD has increased from 18.7 to 28.2% in 2017, and frequency of this delivery method continues on an upward trend [1, 2]. Globally, rates of cesarean sections are considered high at an estimated 21% of livebirths in 2015, based on data from 169 countries [3].

A number of factors influencing the increase of this surgical delivery method include changes in healthcare practice styles, patient preferences, pressures of malpractice and demographic influences (e.g., social, economic, cultural) [4–10]. These influences can affect delivery options/choice and may result in complex pregnancies that ultimately require a CD [11, 12]. Short- and long-term morbidity risks for the infant and mother are further influenced by the etiology or indication of their CD; however, overall risk of morbidity and mortality is more positively associated with CD compared to vaginal delivery [13–16]. This risk warrants careful consideration of potential post-operative complications before scheduled CD, a major abdominal surgery. Recent ‘Early Recovery After Cesarean’ (ERAS) for CD guidelines (Part 1–3) have been published to reduce maternal and neonatal morbidity and mortality [17].

For women who have undergone a prior CD, there is uncertainty regarding the choice of a repeat/scheduled CD or attempting a vaginal delivery for a successive pregnancy as both modes of birth have risks. The Society of Obstetricians and Gynaecologists of Canada (SOGC), the American College of Obstetricians and Gynecologists (ACOG) and the Royal College of Obstetricians and Gynaecologists (RCOG) recommend that a trial of labor be offered to women with one previous transverse low-segment CD [18–20]. Vaginal birth after cesarean (VBAC) may be desired by some women, but the patient-level benefits associated with VBAC including avoiding repeat abdominal surgery and risk of complications in future pregnancies must be considered against the potential risks of a failed trial of labor after cesarean (TOLAC) with subsequent maternal and neonatal morbidity, including an unplanned repeat CD [18]. While the risk for uterine rupture of the previous cesarean incision scar is low (single CD 0.72%; double CD 1.59%) there is maternal and neonatal risk [21].

Many studies have examined factors that are associated with a greater likelihood of a successful TOLAC, commonly identifying a history of successful vaginal delivery [22–24] and women who present in spontaneous labor [19, 22] as significant predictors.

Due to high global cesarean rates, the promotion of VBAC may be one option to reduce the overall number of cesarean deliveries. Clinical interventions that positively impact the rate of vaginal deliveries for women choosing a VBAC need to be examined. This systematic review aimed to synthesize and evaluate the research on clinical interventions that could be directed at or used by patients, families, healthcare providers, and hospitals/ health systems to influence the success of VBAC. A systematic review of ‘adjunct’ clinical interventions that influence the uptake and success of VBAC has been completed and published [4].

## Methods

This study followed standardized methods and guidelines for systematic reviews [25, 26], and used an a priori protocol (available from authors).

### Literature search

A research librarian searched the following databases in May 2017: Ovid Medline (1946-), Ovid Embase (1980-), Wiley Cochrane Library (inception-), CINAHL via EBS-Cohost (1937-) and Ovid PsycINFO (1806-). Limits were applied for language (English and French) and publication year (1985). Update searches were done in November 2018 only in databases from which the included studies were found (Medline and Embase). The search strategy used the Cochrane Proceeding Citation Indexes (Clarivate Analytics) and hand-searched meeting abstracts (2015–2017) from the following associations: The Society for Maternal-Fetal Medicine (SMFM), the Society of Obstetricians and Gynaecologists of Canada (SOGC), and the American Congress of Obstetricians and Gynecologists. Finally, we searched ClinicalTrials.gov and ProQuest Dissertations & Theses Global (1861-). Reference lists of relevant systematic reviews were reviewed for potentially eligible studies. The detailed search strategy is in Additional file 1: Appendix 1.

### Eligibility criteria

The study population was women who had a previous CD including women with more than one prior CD. Births attended by any healthcare provider (e.g., family physician, midwife, obstetrician/gynecologist) were eligible. Any clinical intervention or exposure that was intended to achieve a successful VBAC among women with a prior CD were eligible for inclusion. Studies had to report on at least one of the following pre-determined outcomes: our primary outcome was rate of VBAC among women who attempted a vaginal delivery; secondary outcomes included uterine rupture rates and uterine dehiscence rates, whenever these were reported, as these are considered as being significantly associated with increased likelihood of maternal and neonatal morbidity. Studies that examined deliveries in any setting (e.g., hospitals, primary care centers, birthing units, home births) were eligible. All study designs (randomized [RCT] and non-randomized controlled trials [NRCT], and observational studies) with a comparison group were eligible for inclusion. Studies were not considered eligible if: all women had three or more prior cesareans; multiple births of three or more fetuses were explicitly included; there was an absence of an exposure or intervention, or an inappropriate exposure/intervention was used (e.g., prediction models, pelvimetry, non-clinical interventions such as guidelines for providers); there was absence of a comparator, or an inappropriate comparator was used (e.g., no data for comparison groups in before-after study designs, women without a previous CD); VBAC rates were not reported; or, they were not primary research (e.g., letter, editorial, commentary). Systematic reviews were not included; reference lists therein were screened for potentially relevant studies.

### Study selection

Two reviewers (CJ and AW) independently screened titles and abstracts using a priori eligibility criteria. Full texts of potentially relevant publications were retrieved and independently reviewed in duplicate for inclusion; disagreements were resolved through discussion or third-reviewer (MS) consultation.

### Data extraction

One reviewer extracted data and another verified data from each included study using a pre-specified and piloted form. Data were extracted for relevant study characteristics (design features), population (number of previous cesarean deliveries, parity), intervention, comparator, outcome (VBAC rate [the number of women with a previous CD who undergo a successful vaginal delivery]; uterine rupture rate [the number of women who experience a uterine rupture among those who attempt a vaginal delivery]; and, uterine dehiscence rate [the number of women who experience a uterine dehiscence among those who attempt a vaginal delivery]), funding source, and setting.

Intention-to-treat results were extracted from individual studies whenever possible. For dichotomous data (rates of VBAC, uterine rupture and uterine dehiscence), we reported counts or proportions, and sample size, by study arm. Results of statistical tests (e.g., *p*-values) or summary statistics (e.g., odds ratio [OR], risk ratio [RR], with confidence intervals [CI]) were extracted whenever these were reported within the studies.

### Assessment of methodological quality

Two reviewers (CJ and AW) independently assessed the methodological quality of included studies; disagreements were resolved via consensus. All studies were assessed using the Mixed Methods Appraisal Tool (MMAT) [27].

### Data synthesis

For rates of VBAC, we reported a relative risk and statistically pooled these using the DerSimonian and Laird random effects model with Mantel-Haenszel weights and corresponding 95% CIs. Risk difference (RD) was used for rare outcomes with small event rates (uterine rupture and uterine dehiscence). Statistical heterogeneity was quantified using the I-squared statistic.

Decisions to pool studies were based on comparability of clinical (e.g., treatment) and methodological (i.e., study design) characteristics across studies.

Analyses were performed using Review Manager Version 5.3 [28].

### Assessment of overall certainty of evidence

Two reviewers (MS and AW) assessed the certainty of the body of evidence for each outcome using the Grading of Recommendations Assessment, Development and Evaluation (GRADE) [29], with disagreements resolved through discussion or consultation with a third reviewer (LH). Certainty was assigned initially as high for evidence from trials and low for evidence from observational studies. Each of five domains was then assessed for potential downgrading: study limitations/risk of bias, inconsistency, indirectness, imprecision, and publication bias. An overall score was determined for each outcome using the GRADE certainty of evidence categories: high, moderate, low or very low.

## Results

**Fig. 1:**
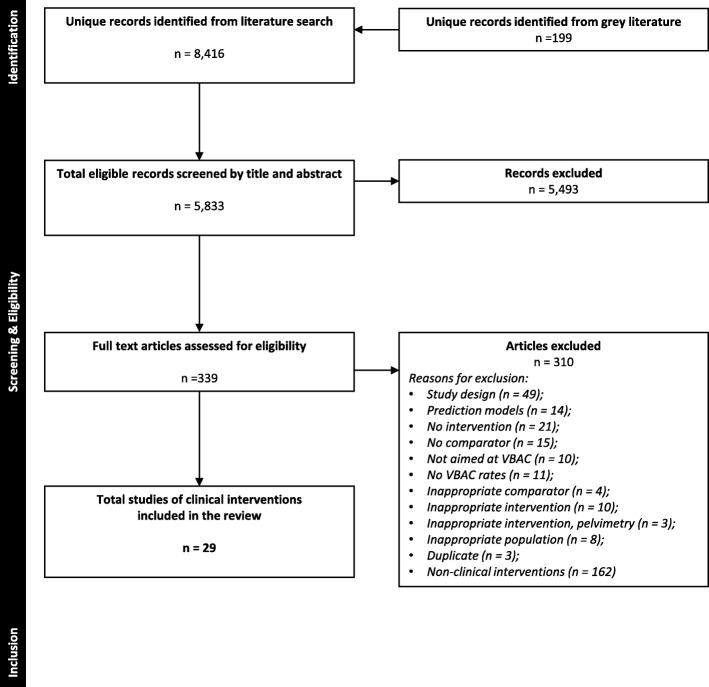
PRISMA flow of study selection

The literature search yielded 5833 unique records. After screening titles and abstracts, 339 potentially relevant articles were identified. Full text screening identified 29 relevant studies [30–58]. The screening process is illustrated in Fig. 1. Table 1 provides a summary of the included studies; detailed study characteristics are in Additional file 1: Appendix 2.

**Table 1:** Summary of included studies

Publication year	median 2003 (range 1984–2017)
Country	N (%)
France, Germany, Hong Kong, Kazakhstan, Morocco, Mozambique, Oman, Poland, Saudi Arabia	1, each (31%)
India, UK	2, each (14%)
Israel	3 (10%)
US	13 (45%)
Funding	N (%)
Non-industry funded	4 (14%)
Industry-funded	1 (3%)
No funding	2 (7%)
NR	22 (76%)
Study design	N (%)
RCT	6 (21%)
Cohort, prospective	12 (41%)
Cohort, retrospective	11 (38%)
Study sample size	Median 237 (range 32–12,676)
Maternal age^a^	Range 17-45y
Proportion of women with any prior vaginal delivery^b^	Range 12–64%
Interventions - pharmacologic	N (%)
Pharmacologic (all)	19 (66%)
PGE2 vs. no PGE2	1 (3%)
PGE1 vs. spontaneous labor	2 (6%)
PGE2 vs. spontaneous labor	3 (10%)
PGE2 vs. expectant management	1 (3%)
Oxytocin vs. no oxytocin	2 (7%)
Oxytocin (AUG) vs. expectant management	1 (3%)
Oxytocin vs. PGE2	1 (3%)
Oxytocin vs. PGE2 vs. spontaneous labor	1 (3%)
Oxytocin (IND) vs. oxytocin (AUG) vs. no oxytocin	3 (10%)
Oxytocin vs. (PGE1 + oxytocin [AUG]) vs. (PGE2 + oxytocin [AUG]) vs. spontaneous labor	1 (3%)
Oxytocin vs. prostanglandin (NS) vs. (oxytocin+prostaglandin [NS]) vs. amniotomy vs. spontaneous labor	1 (3%)
Mifepristone +/− prostaglandin (NS) +/− (amniotomy+oxytocin+epidural) vs. placebo	1 (3%)
Epidural analgesia vs. no epidural analgesia	1 (3%)
Interventions – non-pharmacologic	N (%)
Non-pharmacologic (all)	3 (10%)
Membrane sweep vs. spontaneous labor/no intervention	2 (6%)
Foley catheter 30 mL vs. Foley catheter 80 mL	1 (3%)
Interventions – pharmacologic +/−non-pharmacologic	N (%)
Pharmacologic +/− non-pharmacologic (all)	7 (24%)
Oxytocin vs. (PGE1 +/− amniotomy [IND/AUG]) vs. spontaneous labor	1 (3%)
Foley catheter vs. oxytocin vs. (Foley+oxytocin) vs. PGE2 vs. spontaneous labor	1 (3%)
Foley catheter +/− oxytocin +/− PGE2 +/− amniotomy vs. spontaneous labor	1 (3%)
Oxytocin +/− PGE1 +/− Foley catheter vs. expectant management	1 (3%)
PGE2 vs. PGE2 + balloon catheter	1 (3%)
Oxytocin vs. Cook balloon + oxytocin	1 (3%)
Amniotomy vs. oxytocin vs. PGE1 vs. spontaneous labor	1 (3%)
Outcomes	N (%)
Studies reporting spontaneous onset of labor in addition to VBAC	4 (14%)
Studies reporting assisted vaginal delivery	8 (28%)
Studies reporting uterine dehiscence^c^ only	4 (14%)
Studies reporting uterine dehiscence^c^ and uterine rupture	13 (45%)
Studies reporting uterine rupture	22 (76%)

*NR* not reported, *NS* not specified, *PGE1* prostaglandin E1 (e.g., misoprostol), *PGE2* prostaglandin E2 (e.g., dinoprostone gel, dinoprostone inserts), *RCT* randomized controlled trial, *UK* United Kingdom, *US* United States, *vs* versus, *y* year(s)

^a^ based on studies that provided data on maternal age (*n* = 21)

^b^ based on studies that provided data on prior vaginal delivery (proportion of women in each study arm, *n* = 8)

^c^ uterine dehiscence reported heterogeneously among studies, also includes: asymptomatic dehiscence, threatening uterine rupture, incomplete rupture/dehiscence, uterine scar disruption, uterine scar separation, uterine scar dehiscence, scar dehiscence

All studies included patients who delivered in a healthcare setting.

Studies included a range of 32 to 12,676 women (median 237 women), aged 17 to 45 (among 24 studies). Nine studies (31%) reported parity (range 1 to 12). Among three studies [35, 40, 53] (10%) that reported women with a prior CD, women with a single cesarean comprised the highest proportion of the study population (> 80%), while those with two prior cesarean births (two studies [35, 40]; 650 women) represented approximately 14 to 20%. Eight studies [31, 39, 40, 42, 43, 49, 53, 57] (28%) reported a range of 12 to 64% of women who had a prior or history of vaginal delivery. Approximately 60% women in one study had previously experienced both a cesarean and vaginal delivery [37]. Two studies (7%) reported women with (6% versus 31%, balloon catheter versus oxytocin, respectively) [53], and without (greater than 90%) [49], a history of VBAC. Three studies (10%) reported women with a Bishop’s score at study entry or just prior to labor induction (range 0 to 6) [46, 49, 52]. Six studies [33, 34, 38, 41, 44, 55] (21%) did not report any baseline demographic information for the study population.

Induction of labor is the artificial stimulation of labor before its spontaneous onset to achieve vaginal delivery, taking into account the status/readiness of the cervix (termed “favorable” or “ripe”) prior to initiating the labor process [59]. Cervical ripening or induction of labor can be achieved using non-pharmacologic methods, pharmacologic agents, or some combination of both techniques, each with advantages and disadvantages. Mechanical and surgical methods are forms of non-pharmacologic induction, including membrane stripping, balloon catheters, or amniotomy (artificial rupture of membranes) [60]. Pharmacologic agents commonly used for cervical ripening or induction of labor include prostaglandin (PGE2 analog, in gel or pessary form), misoprostol (PGE1 analog), or mifepristone [60].

All studies examined some manner of cervical ripening and/or labor induction in the intervention group, usually compared to a group of women undergoing spontaneous labor or expectant pregnancy management.

Many cervical ripening and labor induction methods were represented by the included studies.

Twenty-six studies [30–45, 48, 50–58] (90%) examined the effects of at least one pharmacologic agent on VBAC rates, including prostaglandins, oxytocin, mifepristone and epidural analgesia. Ten studies [31, 33, 43, 46–49, 53, 54, 57] (34%) examined non-pharmacologic or mechanical methods (membrane sweeping, amniotomy, balloon devices) in at least one intervention arm.

Studies reported adverse maternal/neonatal outcomes, in addition to VBAC rates, including uterine rupture (10 studies [31, 35, 37, 39, 43, 47, 48, 53, 56, 57]; 34%), uterine dehiscence (four studies [40–42, 55]; 14%), and both uterine rupture and dehiscence (13 studies [30, 32, 34, 36, 38, 44–46, 49–52, 54]; 45%). Eleven (38%) studies provided definitions of uterine rupture, with indications of “disruption of previous scar”, “separation”, “tear” or “rupture” of the uterine wall or peritoneum, and/or extrusion of fetal parts [30, 36, 37, 39, 46, 48, 51, 53, 54, 57, 58].

Of the studies reporting rupture or dehiscence, three [32, 45, 50] (10%) also reported uterine hyper-stimulation and three [41, 42, 44] (10%) also reported uterine atony; these outcomes were not included in analyses as they were considered clinically-related but distinct from the review’s outcomes of interest.

### Methodological quality of included studies (Table 2 and Additional file 1: Appendix 3)

All of the studies reported a clear research question or objective and collected data that addressed the intended research question.

**Table 2:** Summary of methodological quality of included studies

MMAT^a^ criteria	Screening questions		Quantitative/ control group				Quantitative non-randomized				Total
Study	Clear research questions or objectives?	Do collected data address the research questions/objective?	2.1 Clear description of randomization?	2.2 Clear description of allocation concealment (or blinding)?	2.3 Complete outcome data (≥80%)?	2.4 Low withdrawal/drop-out (< 20%)?	3.1 Participants/organizations recruitment - minimizes selection bias?	3.2 Appropriate measurements used for intervention & outcomes?	3.3 Participants/organizations comparable, or are differences accounted for?	3.4 Complete outcome data (80% or above) or acceptable follow-up rate?	
Aboulfalah 2001 (PC) [30]	✰	✰	NA	NA	NA	NA	–	–	✰	✰	✰✰ 50%
Al-Shaikh 2013 (PC) [31]	✰	✰	NA	NA	NA	NA	✰	✰	✰	✰	✰✰✰✰ 100%
Blanco 1992 (PC) [32]	✰	✰	NA	NA	NA	NA	–	✰	✰	✰	✰✰✰ 75%
Cieminski 2015 (RC)	✰	✰	NA	NA	NA	NA	✰	–	–	✰	✰✰ 50%
Cunha 1999 (PC) [34]	✰	✰	NA	NA	NA	NA	–	✰	–	✰	✰✰ 50%
Flamm 1987 (PC) [36]	✰	✰	NA	NA	NA	NA	–	✰	–	✰	✰✰ 50%
Flamm 1997 (PC) [35]	✰	✰	NA	NA	NA	NA	✰	–	–	✰	✰✰ 50%
Geetha 2012 (PC) []37	✰	✰	NA	NA	NA	NA	–	–	✰	✰	✰✰ 50%
Goldman 1998 (PC) [38]	✰	✰	NA	NA	NA	NA	✰	✰	✰	✰	✰✰✰✰ 100%
Grobman 2007 (PC)	✰	✰	NA	NA	NA	NA	✰	–	–	✰	✰✰ 50%
Grubb 1996 (RCT) [40]	✰	✰	✰	–	✰	✰	NA	NA	NA	NA	✰✰✰ 75%
Horenstein 1984 (RC) [42]	✰	✰	NA	NA	NA	NA	✰	✰	✰	✰	✰✰✰✰ 100%
Horenstein 1985 (PC) [41]	✰	✰	NA	NA	NA	NA	✰	✰	–	✰	✰✰✰ 75%
Kehl 2016 (PC) [43]	✰	✰	NA	NA	NA	NA	✰	–	✰	✰	✰✰✰ 75%
Lao 1987 (RC) [44]	✰	✰	NA	NA	NA	NA	✰	✰	–	✰	✰✰✰ 75%
Lelaidier 1994 (RCT) [45]	✰	✰	✰	✰	✰	✰	NA	NA	NA	NA	✰✰✰✰ 100%
Manish 2016 (RCT) [46]	✰	✰	✰	✰	✰	✰	NA	NA	NA	NA	✰✰✰✰ 100%
Ogbonmwan 2010 (RC) [47]	✰	✰	NA	NA	NA	NA	✰	–	–	✰	✰✰ 50%
Palatnik 2015 (RC) [48]	✰	✰	NA	NA	NA	NA	✰	✰	–	✰	✰✰✰ 75%
Ramya 2015 (RCT) [49]	✰	✰	–	✰	✰	✰	NA	NA	NA	NA	✰✰✰ 75%
Rayburn 1999 (RCT) [50]	✰	✰	✰	✰	✰	✰	NA	NA	NA	NA	✰✰✰✰ 100%
Sakala 1990a (RC) [51]	✰	✰	NA	NA	NA	NA	✰	✰	–	✰	✰✰✰ 75%
Sakala 1990b (RC) [52]	✰	✰	NA	NA	NA	NA	✰	–	–	✰	✰✰ 50%
Shah 2017 (RC) [53]	✰	✰	NA	NA	NA	NA	✰	–	–	✰	✰✰ 50%
Shatz 2013 (RC) [54]	✰	✰	NA	NA	NA	NA	✰	✰	–	✰	✰✰✰ 75%
Sims 2001 (RC) [55]	✰	✰	NA	NA	NA	NA	✰	–	–	✰	✰✰ 50%
Taylor 1993 (RCT) [56]	✰	✰	–	✰	✰	✰	NA	NA	NA	NA	✰✰✰ 75%
Tussupkaliyer 2016 (PC) [57]	✰	✰	NA	NA	NA	NA	–	–	✰	✰	✰✰ 50%
Yogev 2004 (RC) [58]	✰	✰	NA	NA	NA	NA	✰	–	✰	✰	✰✰✰ 75%

*PC* prospective cohort, *RC* retrospective cohort, *RCT* randomized controlled trial, *NA* not applicable

^a^ Assessed using the Mixed Methods Appraisal Tool, Version 2011

^✝^Total score is out of 4 stars (✰✰✰✰) for controlled randomized quantitative studies (2.1 to 2.4) and quantitative non-randomized studies (3.1 to 3.4), whereby each assessment criterion met by a study was awarded a star (✰), and a criterion not met by a study was marked with a dash (−);

Of the six RCTs: five [40, 45, 46, 49, 50] (83%) reported a clear description of randomization and of allocation concealment; all reported adequate outcome data (i.e., outcome data available for at least 80% of women) and had low withdrawals or drop-outs (i.e., fewer than 20% of women dropped out of the study) [40, 45, 46, 49, 50, 56].

Of the 23 cohort studies: 17 [31, 33, 35, 38, 39, 41–44, 47, 48, 51–55, 58] (74%) recruited participants in a manner that minimized selection bias; 11 [31, 32, 34, 36, 38, 41, 42, 44, 48, 51, 54] (48%) used appropriate measurements for intervention(s) and outcomes; and, nine [30–32, 37, 38, 42, 43, 57, 58] (39%) ensured that participants were comparable between groups at the beginning of the study or accounted for differences. All studies reported adequate data for the primary outcome of VBAC rates.

### VBAC rates

Effect estimates for VBAC rates are summarized in Table 3. There was low to very low certainty of evidence for all comparisons involving pharmacologic, non-pharmacologic, and combined (pharmacologic and non-pharmacologic) induction methods.

**Table 3:** Effect estimates and certainty of evidence for clinical interventions that influence VBAC rates

Comparison	Study design (no. of studies)	Vaginal delivery – assisted vs. unassisted	Comparison 1 (no. of women with VBAC/no. of women)	Comparison 2 (no. of women with VBAC/no. of women)	Risk Ratio, M-H, Random (95% CI)	I^2^ (%)	Certainty of Evidence
**Pharmacologic induction**							
PGE1 vs. spontaneous labor	Prospective cohort (3)		150/206	343/453	1.08 (0.67, 1.75)	92	Very Low
	Retrospective cohort (1)		21/30	156/167	0.75 (0.59, 0.95)	NA	Very Low
PGE2 vs. spontaneous labor	RCT (1)		82/143	83/151	1.04 (0.85, 1.28)	NA	Low
		Assisted	12/143	9/151	1.41 (0.61, 3.24)	NA	Low
		Unassisted	70/143	74/151	1.00 (0.79, 1.26)	NA	Low
	Prospective cohort (4)		215/314	820/1253	0.98 (0.87, 1.10)	32	Very Low
		Assisted	4/97	60/931	0.64 (0.24, 1.72)	NA	Very Low
		Unassisted	58/97	524/931	1.06 (0.89, 1.26)	NA	Very Low
	Retrospective cohort (1)		37/54	3111/4263	0.94 (0.78, 1.13)	NA	Very Low
Oxytocin vs. spontaneous labor/no oxytocin	Prospective cohort (2)		509/774	1400/1734	0.80 (0.76, 0.85)	0	Very Low
	Retrospective cohort (6)		539/771	3785/5090	0.88 (0.72, 1.07)	91	Very Low
PGE1 + oxytocin vs. spontaneous labor	Retrospective cohort (1)		73/113	156/167	0.69 (0.60, 0.80)	NA	Very Low
Mifepristone vs. placebo	RCT (1)		11/16	8/16	1.38 (0.76, 2.48)	NA	Very Low
		Assisted	5/16	4/16	1.25 (0.41, 3.82)	NA	Very Low
		Unassisted	6/16	4/16	1.50 (0.52, 4.32)	NA	Very Low
Epidural analgesia vs. no epidural	Retrospective cohort (1)		77/87	125/150	1.06 (0.96, 1.18)	NA	Very Low
Oxytocin (induction) vs. oxytocin (augmentation)	Prospective cohort (1)		23/32	177/257	1.04 (0.83, 1.32)	NA	Very Low
	Retrospective cohort (1)		28/48	22/25	0.66 (0.50, 0.88)	NA	Very Low
Active inpatient management (+/− oxytocin) vs. expectant outpatient management (+/− oxytocin)	RCT (1)		80/95	77/93	1.02 (0.90, 1.15)	NA	Very Low
		Assisted	17/95	19/93	0.88 (0.49, 1.58)	NA	Very Low
		Unassisted	63/95	58/93	1.06 (0.86, 1.32)	NA	Very Low
Oxytocin vs. PGE1	Retrospective cohort (1)		52/83	21/30	0.90 (0.67, 1.19)	NA	Very Low
Oxytocin vs. PGE2	RCT (1)		15/21	17/21	0.88 (0.63, 1.24)	NA	Very Low
		Assisted	4/21	5/21	0.80 (0.25, 2.57)	NA	Very Low
		Unassisted	11/21	12/21	0.92 (0.53, 1.59)	NA	Very Low
	Prospective cohort (1)		135/208	105/146	0.90 (0.78, 1.04)	NA	Very Low
	Retrospective cohort (1)		183/254	37/54	1.05 (0.86, 1.28)	NA	Very Low
PGE2 vs. no PGE2	Prospective cohort (1)		233/453	3513/4569	0.67 (0.61, 0.73)	NA	Very Low
Induction (oxytocin, misoprostol + oxytocin augmentation, PGE2) vs. spontaneous labor	Retrospective cohort (1)		33/57	138/179	0.75 (0.59, 0.95)	NA	Very Low
**Non-pharmacologic induction**							
Foley catheter vs. spontaneous labor	Retrospective cohort (1)		221/375	3111/4263	0.81 (0.74, 0.88)	NA	Very Low
Membrane sweep vs. no membrane sweep/spontaneous labor	RCT (1)		13/75	14/75	0.93 (0.47, 1.84)	NA	Very Low
	Retrospective cohort (1)		31/62	49/79	0.81 (0.60, 1.09)	NA	Very Low
		Assisted	13/62	19/79	0.87 (0.47, 1.62)	NA	Very Low
		Unassisted	18/62	30/79	0.76 (0.47, 1.24)	NA	Very Low
Amniotomy vs. spontaneous labor	Prospective cohort (1)		39/62	65/96	0.93 (0.73, 1.18)	NA	Very Low
	Retrospective cohort (1)		477/575	3111/4263	1.14 (1.09, 1.18)	NA	Very Low
30 mL Foley catheter vs. 80 mL Foley catheter	RCT (1)		18/77	15/77	1.20 (0.65, 2.20)	NA	Low
		Assisted	12/77	11/77	1.09 (0.51, 2.32)	NA	Low
		Unassisted	6/77	4/77	1.50 (0.44, 5.11)	NA	Low
Foley catheter vs. Amniotomy	Retrospective cohort (1)		221/375	477/575	0.71 (0.65, 0.78)	NA	Very Low
**Pharmacologic vs. Non-pharmacologic or Combined (pharmacologic + non-pharmacologic)**							
Induction (Foley catheter, PGE2 or oxytocin) vs. spontaneous labor	Prospective cohort (1)		33/52	193/268	0.88 (0.71, 1.10)	NA	Very Low
Induction (oxytocin, prostaglandin or amniotomy) vs. spontaneous labor	Prospective cohort (1)		2165/3259	6477/8519	0.87 (0.85, 0.90)	NA	Very Low
Induction (oxytocin, prostaglandin, Foley catheter +/− surgical) vs. spontaneous labor	Retrospective cohort (1)		1062/1576	3111/4263	0.92 (0.89, 0.96)	NA	Very Low
Oxytocin vs. Foley catheter	Retrospective cohort (1)		183/254	221/375	1.22 (1.09, 1.37)	NA	Very Low
Oxytocin vs. Amniotomy	Retrospective cohort (1)		183/254	477/575	0.87 (0.80, 0.95)	NA	Very Low
Oxytocin vs. Cook balloon + oxytocin	Retrospective cohort (1)		106/150	32/64	1.41 (1.08, 1.84)	NA	Very Low
PGE2 vs. Foley catheter	Retrospective cohort (1)		37/54	221/375	1.16 (0.95, 1.42)	NA	Very Low
PGE2 vs. Amniotomy	Retrospective cohort (1)		37/54	477/575	0.83 (0.69, 0.99)	NA	Very Low
Double-balloon catheter + PGE2 vs. PGE2	Prospective cohort (1)		57/98	71/112	0.92 (0.74, 1.14)	NA	Very Low
		Assisted	10/98	13/112	0.88 (0.40, 1.92)	NA	Very Low
		Unassisted	47/98	58/112	0.93 (0.70, 1.22)	NA	Very Low
Oxytocin +/− amniotomy vs. amniotomy	Retrospective cohort (1)		86/102	26/35	1.13 (0.92, 1.40)	NA	Very Low
		Assisted	17/102	4/35	1.46 (0.53, 4.04)	NA	Very Low
		Unassisted	69/102	22/35	1.08 (0.81, 1.44)	NA	Very Low
> 1 induction method vs. 1 induction method	Retrospective cohort (1)		142/314	920/1259	0.62 (0.55, 0.70)	NA	Very Low
Induction (Oxytocin +/− Foley catheter +/− PGE1) vs. expectant management	Retrospective cohort (1)		1088/1631	6787/11045	1.09 (1.05, 1.13)	NA	Very Low
Induction vs. no induction - Women with prior VD	Prospective cohort (3) Retrospective cohort (1)		1383/1667	3802/4315	0.94 (0.92, 0.97)	0	Very Low
Induction vs. no induction - Women without prior VD	Prospective cohort (3) Retrospective cohort (1)		1040/1980	3336/4970	0.75 (0.69, 0.81)	35	Very Low

Risk ratios that are statistically significant have been bolded

*CI* confidence interval, *NA* not applicable, *NS* not specified, *no.* number, *PGE1/PGE2* prostaglandin 1/prostaglandin 2, *RCT* randomized clinical trial, *VBAC* vaginal birth after cesarean, *VD* vaginal delivery, *vs.* versus

#### Pharmacologic induction

Nineteen (66%) studies compared a pharmacologic intervention to spontaneous labor, no intervention, or another pharmacologic agent. Many comparators were reported by a single study. Four cohort studies comparing PGE1 to spontaneous labor showed no significant differences between groups on VBAC rates (three prospective cohorts [30, 34, 57], RR 1.08, 95% CI 0.67 to 1.75; I^2^ = 92%; and, one retrospective cohort [33], RR 0.75, 95% CI 0.59 to 0.95). One RCT of PGE2 compared to spontaneous labor found no significant differences between groups (RR 1.04, 95% CI 0.85 to 1.28) [50]. One study found increased rates of VBAC among women not induced versus induction using PGE2 (RR 0.67, 95% CI 0.61 to 0.73) [35]. Four prospective cohort studies found no significant differences between PGE2 and spontaneous labor on rates of VBAC (RR 0.98, 95% CI 0.87 to 1.10; I^2^ = 32) [32, 37, 38, 58]. One retrospective cohort comparing an PGE2 with spontaneous labor found no significant differences in VBAC rates (RR 0.94, 95% CI 0.78 to 1.13) [54]. Increased VBACs among women with spontaneous labor compared with oxytocin were found in two studies (RR 0.80, 95% CI 0.76 to 0.85; I^2^ = 0%) [36, 41] but no significant differences were reported between oxytocin and spontaneous labor from six retrospective cohorts [33, 38, 42, 51, 54, 57] (RR 0.88, 95% CI 0.72 to 1.07, I^2^ = 91%).

One retrospective cohort found increased rates of VBAC among women undergoing spontaneous labor versus women induced with PGE1 and oxytocin (RR 0.69, 95% CI 0.60 to 0.80) [33]. Two studies showed conflicting results comparing oxytocin for induction to oxytocin for augmentation (one prospective cohort [41], RR 1.04, 95% CI 0.83 to 1.32; one retrospective cohort [51], RR 0.66, 95% CI 0.50 to 0.88). No significant differences were found for oxytocin compared to PGE1 (one retrospective cohort [33], RR 0.90, 95% CI 0.67 to 1.19). No significant differences in VBAC rates between oxytocin and PGE2 were found in one RCT [56] (RR 0.88, 95% CI 0.63 to 1.24), one prospective cohort [38] (RR 0.90, 95% CI 0.78 to 1.04) and one retrospective cohort [54] (RR 1.05, 95% CI 0.86 to 1.28). One RCT of mifepristone versus placebo found no significant difference in VBAC rates (RR 1.38, 95% CI 0.76 to 2.48) [45]. A retrospective cohort found no significant differences in VBACs for women with versus without epidural analgesia (RR 1.06, 95% CI 0.96 to 1.18) [52]. Increased VBACs were found among women undergoing spontaneous labor compared with women induced with multiple pharmacologic agents (PGE2, misoprostol with oxytocin augmentation, and oxytocin alone) (RR 0.75, 95% CI 0.59 to 0.95) [55]. One RCT of active inpatient management compared to expectant management (both groups had some women with and without oxytocin) found no significant differences in VBAC rates (RR 1.02, 95% CI 0.90 to 1.15) [40].

#### Non-pharmacologic induction

Five (17%) studies compared a mechanical induction modality to spontaneous labor or no intervention, or compared two different mechanical induction methods [46, 47, 49, 54, 57]. One RCT found no significant differences in VBAC rates between 30 mL and 80 mL Foley catheters (RR 1.20, 95% CI 0.65 to 2.20) [46]. Another RCT found for no significant differences in VBACs among women induced with versus without membrane sweeping (RR 0.93, 95% CI 0.47 to 1.84) [49]. Increased rates of VBAC were seen among women undergoing spontaneous labor compared with women induced with a Foley catheter (RR 0.81, 95% CI 0.74 to 0.88) [54]. The same retrospective cohort found higher rates of VBAC among women induced with amniotomy versus Foley catheter (RR 0.71, 95% CI 0.65 to 0.78) [54]. No significant differences in VBAC rates were found between membrane sweep induction in a retrospective cohort (RR 0.81, 95% CI 0.60 to 1.09) [47], or amniotomy in two cohorts (RR 1.06, 95% CI 0.88 to 1.28) compared with spontaneous labor [54, 57].

#### Pharmacologic and non-pharmacologic (combined)

Seven (24%) studies compared one arm of combined pharmacologic and non-pharmacologic induction methods [30, 39, 43, 44, 48, 53, 54]. One cohort study found increased rates of VBAC among women undergoing spontaneous labor compared with women induced with any of prostaglandin (unspecified analog), oxytocin or amniotomy (RR 0.87, 95% CI 0.85 to 0.90) [39]. One cohort study found increased rates of VBAC among women undergoing expectant management compared with women induced with any of oxytocin, Foley catheter and/or PGE1 (RR 0.1.09, 95% CI 1.05 to 1.13) [48]. No significant differences in VBAC rates were found between any of PGE2, oxytocin or Foley catheter with spontaneous labor in one cohort study (RR 0.88, 95% CI 0.71 to 1.10) [31]. One cohort study found increased VBACs among women induced with Cook balloon and oxytocin (combined) versus oxytocin only (RR1.41, 95% CI 1.08 to 1.84) [53]. Two cohort studies (*n* = 3) comparing pharmacologic and mechanical (combined) with either pharmacologic or mechanical induction methods found no significant differences on rates of VBAC: double-balloon catheter and PGE2 versus PGE2 only (RR 0.92, 95% CI 0.74 to 1.14) [43]; and, amniotomy and oxytocin versus amniotomy only (RR 1.13, 95% CI 0.92 to 1.40) [44]. One retrospective cohort comparing multiple with a single induction method found increased VBAC rates for women induced with a single method (RR 0.62, 95% CI 0.55 to 0.70) [54].

#### Pharmacologic versus non-pharmacologic

One cohort study compared pharmacologic with mechanical induction methods [54]. No significant differences in VBAC rates were found between PGE2 and Foley catheter (RR 1.16, 95% CI 0.95 to 1.42) [54]. Women induced surgically (amniotomy) had increased VBAC rates compared with women induced with PGE2 (RR 0.83, 95% CI 0.69 to 0.99) or with oxytocin (RR 0.87, 95% CI 0.80 to 0.95) but no significant differences were found between oxytocin and Foley catheter (RR 1.22, 95% CI 1.09 to 1.37) [54].

#### Assisted versus unassisted vaginal deliveries

Several studies stratified VBAC rates according to assisted versus unassisted (spontaneous) vaginal delivery. Among the RCTs, there were no significant differences in one trial [50] comparing PGE2 with spontaneous labor (assisted, RR 1.41, 95% CI 0.61 to 3.24 versus unassisted, RR 1.00, 95% CI 0.79 to 1.26) and another trial [46] comparing 30 mL Foley catheter with 80 mL Foley catheter (assisted, RR 1.09, 95% CI 0.51 to 2.32 versus unassisted, RR 1.50, 95% CI 0.44 to 5.11). No significant differences between groups were found in other RCTs [40, 45, 56] and cohort studies [44, 47, 58] (various comparisons) that stratified vaginal deliveries as assisted or unassisted (very low certainty of evidence).

Among five cohort studies [37, 39, 41–43] reporting VBAC rates for women with and without a prior vaginal delivery, four that compared induction with no induction were pooled. Results showed increased rates of VBAC among women with a prior vaginal delivery whose labor was not induced (RR 0.94, 95% CI 0.92 to 0.97; I^2^ = 0%), and for women without a prior vaginal delivery whose labor was not induced (RR 0.75, 95% CI 0.69 to 0.81; I^2^ = 35%) [37, 39, 41, 42].

### Uterine rupture rates

Of the studies that reported on uterine rupture, most had zero cases among study arms. Effect estimates are summarized in Table 4. All comparisons for pharmacologic, non-pharmacologic, combined (pharmacologic and non-pharmacologic) induction methods provided low to very low certainty of evidence.

**Table 4:** Effect estimates and quality of evidence for clinical interventions that influence uterine rupture rates

Comparison	No. of studies	Comparison 1 (no. of women with uterine rupture/no. of women)	Comparison 2 (no. of women with uterine rupture/no. of women)	Risk Difference, M-H, Random (95% CI)	I^2^ (%)	Certainty of Evidence
Pharmacologic induction						
PGE1 vs. spontaneous labor	Prospective cohort (2)	3/117	21/357	-0.01 (− 0.10, 0.08)	83	Very Low
PGE2 vs. no PGE2/spontaneous labor	RCT (1)	0/143	0/151	0.00 (− 0.01, 0.01)	NA	Low
	Prospective cohort (6)	7/819	38/6090	0.00 (0.00, 0.01)	0	Very Low
Oxytocin vs. spontaneous labor/no oxytocin	Prospective cohort (2) Retrospective cohort (1)	2/766	1/1621	0.00 (0.00, 0.01)	0	Very Low
Epidural analgesia vs. no epidural	Retrospective cohort (1)	0/87	0/150	0.00 (− 0.02, 0.02)	NA	Very Low
Mifepristone vs. placebo	RCT (1)	0/16	0/16	0.00 (− 0.11, 0.11)	NA	Low
Oxytocin vs. PGE2	Prospective cohort (1)	0/208	0/146	0.00 (− 0.01, 0.01)	NA	Very Low
Non-pharmacologic induction						
Membrane sweep vs. spontaneous labor	RCT (1)	0/62	0/61	0.00 (− 0.03, 0.03)	NA	Very Low
	Retrospective cohort (1)	0/62	0/79	0.00 (− 0.03, 0.03)	NA	Very Low
Pharmacologic vs. Non-pharmacologic or Combined (pharmacologic + non-pharmacologic)						
Oxytocin + amniotomy vs. PGE2 + amniotomy	RCT (1)	0/21	1/21	-0.05 (− 0.17, 0.07)	NA	Very Low
Oxytocin vs. Cook balloon + oxytocin	Retrospective cohort (1)	2/150	0/64	0.01 (− 0.02, 0.04)	NA	Very Low
Double-balloon catheter + PGE2 vs. PGE2	Prospective cohort (1)	0/98	1/112	-0.01 (− 0.03, 0.02)	NA	Very Low
30 mL Foley catheter vs. 80 mL Foley catheter	RCT (1)	1/77	1/77	0.00 (−0.04, 0.04)	NA	Low
Induction (oxytocin, prostaglandin, oxytocin + prostaglandin, or amniotomy) vs. spontaneous labor	Prospective cohort (1)	35/3259	54/8519	0.00 (0.00, 0.01)	NA	Very Low
Induction (oxytocin +/− PGE1 +/− Foley catheter) vs. expectant management	Retrospective cohort (1)	22/1631	59/11045	0.01 (0.00, 0.01)	NA	Very Low

*CI* confidence interval, *NA* not applicable, *NS* not specified, *no.* number, *PGE1/PGE2* prostaglandin 1/prostaglandin 2; *RCT* randomized clinical trial, *VD* vaginal delivery, *vs.* versus

#### Pharmacologic induction

One RCT reported no cases of uterine rupture in either group comparing PGE2 with no induction (RD 0.00, 95% CI − 0.01 to 0.01) [50]. A small RCT (*n* = 16) comparing mifepristone with placebo also found no significant differences between groups (RD 0.00, 95% CI − 0.11 to 0.11) [45]. All other pharmacologic induction agents compared with spontaneous labor or no intervention (all cohort studies) on rates of uterine rupture found no significant differences: PGE1 (two studies [30, 34], RD -0.01, 95% CI − 0.10 to 0.08; I^2^ = 83%), PGE2 (six studies [31, 32, 35, 37, 38, 58], RD 0.00, 95% CI 0.00 to 0.01; I^2^ = 0%), and oxytocin (3 studies [36, 38, 51], RD 0.00, 95% CI 0.00 to 0.01; I^2^ = 0%). No significant differences in uterine ruptures were found among single cohort studies comparing epidural analgesia with no intervention (zero events; RD 0.00, 95% CI − 0.02 to 0.02) [52], and oxytocin with PGE2 (RD 0.00, 95% CI − 0.01 to 0.01) [38].

#### Non-pharmacologic induction

One RCT found no significant differences in uterine ruptures between induction with Foley catheters (30 mL versus 80 mL) (RD 0.00, 95% CI − 0.04 to 0.04) [46]. No significant differences between membrane sweeping and spontaneous labor were found from one RCT [49] (RD 0.00, 95% -0.03, 0.03) and one cohort study [47] (RD 0.00, 95% CI − 0.03 to 0.03).

#### Pharmacologic and non-pharmacologic (combined)

One RCT found no significant differences between induction with oxytocin (plus amniotomy) and PGE2 (plus amniotomy) (RD -0.05, 95% CI − 0.17 to 0.07) [56]. One cohort study found no significant differences between induction (any of oxytocin, prostaglandin, oxytocin and prostaglandin [combined], or amniotomy) or spontaneous labor (RR 0.00, 95% CI 0.00 to 0.01) [39]. Another cohort study found no significant differences between induction with any of oxytocin, Foley catheter and/or PGE1 compared with expectant management (RD 0.01, 95% CI 0.00 to 0.01) [48]. No significant differences in uterine ruptures for women induced with oxytocin compared with women induced with oxytocin and Cook’s balloon (RD 0.01, 95% CI − 0.02 to 0.04) [53]. No significant differences were found between PGE2 and PGE2 with double-balloon catheter (combined) in one cohort study (RD -0.01, 95% CI − 0.03 to 0.02) [43].

### Uterine dehiscence rates

Effect estimates for uterine dehiscence rates are summarized in Table 5. All comparisons involving pharmacologic, non-pharmacologic and combined (pharmacologic and non-pharmacologic) induction methods provided low to very low certainty of evidence.

**Table 5:** Effect estimates and certainty of evidence for clinical interventions that influence uterine dehiscence rates

Comparison	No. of studies	Comparison 1 (no. of women with uterine dehiscence/no. of women)	Comparison 2 (no. of women with uterine dehiscence/no. of women)	Risk Difference, M-H, Random (95% CI)	I^2^ (%)	Quality
Pharmacologic induction						
PGE1 vs. spontaneous labor	Prospective cohort (2)	5/117	6/357	0.02 (−0.06, 0.09)	65	Very Low
PGE2 vs. spontaneous labor	Prospective cohort (2)	1/171	0/222	0.01 (−0.01, 0.02)	0	Very Low
Oxytocin vs. spontaneous labor	Prospective cohort (3) Retrospective cohort (2)	21/1113	16/2298	0.01 (0.00, 0.02)	20	Very Low
Mifepristone vs. placebo	RCT (1)	1/16	1/16	0.00 (− 0.17, 0.17)	NA	Low
Epidural analgesia vs. no epidural	Retrospective cohort (1)	4/87	1/150	0.04 (− 0.01, 0.09)	NA	Very Low
Oxytocin vs. PGE2	Prospective cohort (1)	1/208	1/146	0.00 (−0.02, 0.01)	NA	Very Low
Active inpatient management (+/− oxytocin) vs. expectant outpatient management (+/− oxytocin)	RCT (1)	5/95	0/93	0.05 (0.00, 0.10)	NA	Very Low
Non-pharmacologic induction						
Membrane sweep vs. spontaneous labor	RCT (1)	0/62	1/61	-0.02 (− 0.06, 0.03)	NA	Very Low
30 mL Foley catheter vs. 80 mL Foley catheter	RCT (1)	2/77	7/77	−0.06 (− 0.14, 0.01)	NA	Low
Pharmacologic vs. Non-pharmacologic or Combined (pharmacologic + non-pharmacologic)						
Oxytocin +/− amniotomy vs. amniotomy	Retrospective cohort (1)	4/102	0/35	0.04 (− 0.02, 0.09)	NA	Very Low
Induction (PGE2, oxytocin, amniotomy and/or Foley catheter) vs. spontaneous labor	Retrospective cohort (1)	13/1576	36/4263	0.00 (− 0.01, 0.01)	NA	Very Low
Induction (oxytocin, PGE2, misoprostol + oxytocin [augmentation]) vs. spontaneous labor	Retrospective cohort (1)	4/57	3/179	0.05 (−0.02, 0.12)	NA	Very Low

*CI* confidence interval, *NA* not applicable, *NS* not specified, *no.* number, *PGE1/PGE2* prostaglandin 1/prostaglandin 2; *RCT* randomized clinical trial, *VD* vaginal delivery, *vs.* versus

#### Pharmacologic induction

One small RCT found no differences in cases of uterine dehiscence between mifepristone and placebo (*n* = 32; RD 0.00, 95% CI − 0.17 to 0.17) [45]. No differences were found in uterine dehiscence rates comparing induction (oxytocin, PGE1 or PGE2) and no induction (RD 0.05, 95% CI − 0.02 to 0.12) [55]. No differences were found in rates of uterine dehiscence among women induced with PGE1 (two cohort studies [30, 34], RD 0.02, 95% CI − 0.06 to 0.09), PGE2 (two cohort studies [32, 38], RD 0.01, 95% CI − 0.01 to 0.02) or oxytocin (five cohort studies [36, 38, 41, 42, 51], RD 0.01, 95% CI 0.00 to 0.02), when compared with spontaneous labor. One cohort study found no significant differences in uterine dehiscence rates between women with and without epidural analgesia (RD 0.04, 95% CI − 0.01 to 0.09) [52]. No significant differences were found for uterine dehiscence cases between induction with oxytocin versus PGE2 (one cohort study [38], RD 0.00, 95% CI − 0.02 to 0.01) or amniotomy and oxytocin (combined) versus amniotomy only (one cohort study [44], RD 0.04, 95% CI − 0.02 to 0.09). One RCT found increased rates of uterine dehiscence among women managed actively (as inpatients) compared with women managed expectantly (as outpatients), where some women received oxytocin in both groups (RD 0.05, 95% CI 0.00 to 0.10) [40].

#### Non-pharmacologic induction

One RCT found no differences in uterine dehiscence rates comparing 30 mL and 80 mL Foley catheters (RD -0.06, 95% CI − 0.14 to 0.01) [46]. Another RCT found no difference comparing membrane sweeping with spontaneous labor (one RCT, RD -0.02, 95% CI − 0.06 to 0.03) [49].

#### Pharmacologic and non-pharmacologic (combined)

One cohort study found no significant differences in rates of uterine dehiscence between women with (any of PGE2, oxytocin, amniotomy with or without Foley catheter) and without induction (RD 0.00, 95% CI − 0.01 to 0.01) [54].

## Discussion

Overall, there is low to very low certainty of evidence for clinical interventions that influence rates of VBAC, owing to downgrading mainly for the GRADE domains of risk of bias, inconsistency and imprecision. Many of the comparisons were based on single studies with small sample sizes and event rates. The interventions were heterogeneous and focused on pharmacologic and/or mechanical methods of induction. Most of the evidence showed no significant differences between groups on VBAC rates, with very low certainty of evidence. There was some evidence of higher VBAC rates among women who undergo spontaneous labor, when compared to women whose labors are induced, regardless of method or agent used for induction; however, there was very low certainty in this body of evidence.

Other systematic reviews of clinical interventions have been reported in the literature. Catling-Paull et al examined the effect of clinical interventions on the uptake or success of VBACs and found that inductions of labor by amniotomy, prostaglandins, or oxytocin (or a combination of these methods) were associated with lower rates of vaginal deliveries (six RCTs and 28 cohort studies) [61]. The review also examined prognostic criteria/factors and concluded that pelvimetry may adversely affect women’s chances of a VBAC, scoring systems are not clinically useful, and there is lack of sufficient data to inform the value of assessing methods of cesarean closures for predicting successful vaginal deliveries [61]. A Cochrane systematic review compared women (with a prior CD) undergoing cervical ripening and/or labor induction with placebo, no treatment or other methods, and found overall moderate to low certainty of evidence for the varied interventions represented among the small number of included studies, concluding insufficient availability and high-quality of evidence to determine the optimal method of labor induction [62].

### Strengths and limitations of study

This systematic review of clinical interventions for influencing VBAC rates encompassed a broad range of study designs and strategies for cervical ripening and/or labor induction. The nature of the labor process and difficulty blinding women or healthcare providers to interventions lends few studies to an RCT design. Unsurprisingly, nearly 80% of the studies were cohort designs. However, observational studies have inherent biases due to confounding and there were concerns among the included cohorts regarding comparability of population characteristics between groups. Few studies adequately reported maternal characteristics. Some studies included women with only one CD while others enrolled women with multiple cesarean deliveries, a documented risk of increased uterine rupture among women attempting a VBAC. Additionally, few studies reported parity, type of previous uterine incision, prior vaginal delivery, prior VBAC, or indication(s) for previous CD. The concept of spontaneous labor among most studies is erroneously represented in that women in a comparator group (as opposed to the intervention group) are often managed expectantly, where they may or may not then progress to spontaneous labor [18]. Further, spontaneous onset of labor is often used synonymously with spontaneous delivery. One outcome of induction of labor (versus expectant management) is induction success (or failed induction) distinguished from VBAC success. Ambiguous operationalization of these concepts may result in important differences in outcomes. Among women who were induced, studies often did not provide data for the number of women whose labor was augmented. While VBAC was reported by all studies, other perinatal outcomes were unreported or undefined despite following up women to delivery. Four (14%) studies did not report whether there were cases of uterine ruptures in their study population. Among studies that reported on uterine ruptures and/or uterine dehiscence, authors defined these outcomes inconsistently (and without a cited reference) or they were not explicitly differentiated from each other, or they were undefined altogether. Contextual and mediating factors, such as induction-to-labor interval or co-interventions were sparsely reported for women in all study arms.

### Implications for practice

The ability for the maternity care provider to use this evidence to counsel women with a previous CD so they have the information to make the best choice for their situation is a goal of this systematic review. Ananth et al completed a population based study, in the USA, to show the impact of scheduled/repeat CD on the total number of unscheduled/emergency and scheduled/repeat CD [63]. From 1979 to 2010, unscheduled and scheduled CD increased 68% (95% CI 67–69) and 178% (95% CI 176–179), respectively [63]. For the total number of deliveries in 2010, 18.5 and 14.4% were unscheduled and scheduled CDs, respectively [63]. Maternal factors attributed to the unscheduled cesarean increases were increased maternal age, obesity and chronic hypertension [63]. Clinical impact for the scheduled CD group of 14.4% (likely higher in 2019) requires better VBAC information for maternal education and choice [63].

One of the aspects of this VBAC choice is that women cannot choose to be in spontaneous labor at any given gestational age [64]. In their VBAC choice, women are faced with the choice of expectant pregnancy management (waiting for labor to start) or consider an induction of labor protocol acceptable for VBAC [65] or having the scheduled CD. Cervical status/Bishop score is an important clinical factor for induction of labor. The induction of labor protocol for women with a prior uterine scar reports that oxytocin is the only induction agent that can be used other than mechanical methods [65]. Oxytocin has limited effectiveness for cervical ripening. Adequate cervical dilatation allows for amniotomy and oxytocin use to assist in induction process [59]. There are no ‘real’ expectant management VBAC cohorts. For non-VBAC cohorts, there are studies comparing risks of elective labor induction with expectant management. For multiparous women a large retrospective cohort study showed that induction of labor is associated with decreased pregnancy-related hypertension and increased time in labor and delivery [66]. A Cochrane review compared induction of labor at or beyond term with expectant management in uncomplicated, singleton pregnancies, with no parity factor identified; they reported that induction was associated with lower risk of perinatal death, stillbirth and fewer cesarean section, but more operative vaginal birth and no change for perineal trauma, postpartum hemorrhage, stillbirth, low Apgar score, or neonatal distress [67].

While the studies show heterogeneity and certainty of evidence is low to very low, the uterine rupture and dehiscence rates among studies are very clinically equivalent to rates reported in the literature (ruptures, pharmacological/non-pharmacological/combined for each comparison group 0.56, 0.70%; 0, 0; 1.05, 0.65%, respectively and dehiscence, pharmacological/non-pharmacological/combined for each comparison group 2.10, 0.76%; 1.44, 0.72%; 1.21, 0.87%, respectively). These findings can support the safety of oxytocin use for ripening alone and in combination with mechanical methods to allow amniotomy as commented above.

#### Candidates for TOLAC

Various maternal characteristics, clinical history, and presenting labor indications associated with likelihood of VBAC have been reported in the literature. Factors associated with increased risk of maternal morbidity from a TOLAC include: prior classical cesarean section or T incision as a contraindication [18–20], previous low segment cesarean section where the uterus was closed in a single layer [19], less than 18-month inter-delivery interval [19], medical induction or augmentation of labor as compared to spontaneous labor [20], medical induction of labor with misoprostol [18, 19] or PGE2 [19], and increasing number of prior CDs [19].

A previous successful trial of vaginal delivery is associated with greater likelihood of successful VBAC; alternatively, the history of a failed trial of vaginal delivery requires a CD [15, 20, 68, 69]. Multiple obstetrical indications for inducing labor or considering CD, also need to be considered (e.g., post-dates, hypertension, macrosomia, intra-uterine growth restriction, unfavorable cervix) [20, 70]. Different induction methods may have differential effects on progress of labor, and the impact on mode of delivery. Limited evidence from RCTs on methods of labor induction have not been able to determine which method results in greater benefits and lower risks [62].

Evidence-based guidelines comparison from the US (2010 version), Canada (2005 version) and United Kingdom (2007 version) show similarities and differences in recommendations for VBAC process [71]. Uterine rupture is identified as the most serious complication among women attempting a trial of labor; however, there is recognition that differences in the definition may contribute to the reported incidence rates [71]. With regards to induction of labor, all three guidelines agree that induction and augmentation of labor is an option for women attempting a VBAC, but there is an increased risk of uterine rupture with inappropriate use of oxytocin [71]. Both the American College of Obstetricians and Gynecologists (ACOG) and SOGC recommend against PGE1 for the same outcome, with the SOGC guideline recommending PGE2 only in rare circumstances [71]. All three guidelines agree that women with a history of one or two uncomplicated low-transverse CD and no contraindications to VBAC should be educated, counselled and offered a TOLAC [71]. The SOGC’s updated guidelines (July 2019) continue to support the previous recommendation of offering women (without contraindications) the option of a TOLAC [19]. The new guideline provides details on the likelihood of VBAC (greater among women with spontaneous labor, lower among women with factors that negatively affect this likelihood), the risk of uterine rupture (baseline risk of 0.47% among women with a prior CD; higher relative risk of uterine rupture, but low absolute risks for women attempting TOLAC compared with elective repeat cesarean section; risk greatest for women over 40 weeks of gestation; use of fetal monitoring is recommended as an indicator of the presence of uterine rupture), risk of other outcomes (higher relative risk of maternal and perinatal morbidity, but lower absolute risks for women attempting TOLAC compared with elective repeat cesarean section; higher relative risk of maternal death for elective cesarean section), and recommends against using ultrasonographic measurements of the lower uterine segment to counsel patients on the possibility of a TOLAC [19].

#### Shared decision-making

For women with a previous CD, the decision to proceed with a repeat cesarean section or attempt a vaginal delivery will be based on multiple factors. First steps in the decision-making process should be consultation with a healthcare provider regarding maternal and neonatal benefits and risks, for both VBAC (induction/spontaneous onset of labor) and scheduled CD options. Triaging women for VBAC (i.e., women without contraindications) must include maternal characteristics and obstetric history to assist in the prediction of a successful vaginal delivery. Family planning plays a key role. Women who have had one or more prior cesarean deliveries may opt for a trial of vaginal birth or elect to have another CD; this maternal decision impacts future pregnancies and delivery due to the increased risk of abnormal placentation secondary to the additional uterine surgeries [72]. Unexpected severe adverse maternal and neonatal outcomes (e.g., maternal mortality) are important for patient-level counselling and decision-making.

Each woman should be given the opportunity to be counselled early in pregnancy on her available options, with provision of written or on-line patient information literature (e.g., VBAC clinical care pathway, decision aids) to help inform decision-making throughout her pregnancy for optimal delivery outcomes and patient satisfaction.

## Conclusion

This systematic review evaluated clinical interventions for safe practice directed at increasing the rate of vaginal delivery among women with a prior CD but found low to very low certainty in the body of evidence for cervical ripening and/or labor induction techniques. There is insufficient high-quality evidence to inform on optimal clinical interventions/protocols for women considering to have a VBAC.

## Supplementary information

**Additional file 1: Appendix 1.** Search strategy. **Appendix 2.** Characteristics of included studies. **Appendix 3.** Methodological quality assessments of included studies.

## Data Availability

All data generated or analyzed during this study are included in this published article (and its supplementary information files).

## Abbreviations

ACOG: American College of Obstetrics and Gynecology
CD: Cesarean delivery
CI: Confidence interval
GRADE: Grading of Recommendations Assessment, Development and Evaluation
MMAT: Mixed Methods Appraisal Tool
NRCT: Non-randomized clinical trial
OR: Odds ratio
OR: Odds ratio
PGE1: Prostaglandin E1
PGE2: Prostaglandin E2
RCT: Randomized clinical trial
RR: Risk ratio
SMFM: The Society for Maternal-Fetal Medicine
SOGC: The Society of Obstetricians and Gynaecologists of Canada
TOLAC: Trial of labor after cesarean
VBAC: Vaginal birth after cesarean
