# Supplementary material for: Clinical interventions that influence vaginal birth after cesarean delivery rates: Systematic Review & Meta-Analysis
Source: BMC Pregnancy Childbirth. 2019 Dec 30;19:529. doi: 10.1186/s12884-019-2689-5 (PMC6937863; doi:10.1186/s12884-019-2689-5)
Supplement: Supplementary file 1 — Additional file 1: Appendix 1. Search strategy. Appendix 2. Characteristics of included studies. Appendix 3. Methodological quality assessments of included studies. [file 12884_2019_2689_MOESM1_ESM.pdf]

Appendix I. Search strategy

Database: Ovid MEDLINE(R) Epub Ahead of Print, In-Process & Other Non-Indexed Citations, Ovid MEDLINE(R) Daily and Ovid MEDLINE(R) 1946 to Present
Date search conducted: 19 May 2017
Strategy:
1 Vaginal Birth after Cesarean/ (1420)
2 Trial of Labor/ (1051)
3 TOLAC*.tw,kf. (114)
4 (trial adj2 labo?r).tw,kf. (1119)
5 ((vaginal birth or vaginal delivery) adj2 c?esarean*).tw,kf. (1692)
6 VBAC*.tw,kf. (608)
7 or/1-6 [Combined MeSH & text words for VBAC] (3326)
8 exp animals/ not humans/ (4401774)
9 7 not 8 (3308)
10 limit 9 to (english or french) (3064)
11 limit 10 to yr="1985-Current" (2922)
12 remove duplicates from 11 (2792)

Database: Ovid Embase 1980 to 2017 Week 20
Date search conducted: 19 May 2017
Strategy:
1 "trial of labor"/ (848)
2 vaginal birth after cesarean/ (118)
3 TOLAC*.tw,kw. (249)
4 (trial adj2 labo?r).tw,kw. (1496)
5 ((vaginal birth or vaginal delivery) adj2 c?esarean*).tw,kw. (2244)
6 VBAC*.tw,kw. (934)
7 or/1-6 [Combined Emtree & text words for VBAC] (3687)
8 exp animal/ not human/ (4313786)
9 7 not 8 (3658)
10 limit 9 to (english or french) (3433)
11 limit 10 to yr="1985-Current" (3349)
12 remove duplicates from 11 (3287)

Database: Wiley Cochrane Library
Date search conducted: 19 May 2017
Strategy:
#1 [mh ^"Trial of Labor"] 38
#2 [mh ^"Vaginal Birth after Cesarean"] 57
#3 TOLAC*:ti,ab,kw 11
#4 (trial next/2 labo*):ti,ab,kw 286
#5 (("vaginal birth" or "vaginal delivery") next/2 (caesarean* or cesarean*)):ti,ab,kw 146
#6 VBAC*:ti,ab,kw 36
#7 {or #1-#6} 400
#8 #7 Publication Year from 1985 to 2017 389

Database: CINAHL Plus with Full Text via EBSCOhost
Date search conducted: 19 May 2017
Strategy:

#	Query	Limiters/Expanders	Results
S9	S6 NOT S7	Limiters - Published Date: 19850101-20171231; Language: English, French<br>Search modes - Find all my search terms	1,844
S8	S6 NOT S7	Search modes - Find all my search terms	1,869
S7	(MH "Animals+") NOT (MH "Human")	Search modes - Find all my search terms	65,962
S6	S1 or S2 or S3 or S4 or S5	Search modes - Find all my search terms	1,870
S5	VBAC*	Search modes - Find all my search terms	419
S4	("vaginal birth" or "vaginal delivery") N2 (caesarean* or cesarean*)	Search modes - Find all my search terms	1,641
S3	trial N2 labo#r	Search modes - Find all my search terms	429
S2	TOLAC*	Search modes - Find all my search terms	63
S1	(MH "Vaginal Birth After Cesarean")	Search modes - Find all my search terms	1,135

Database: Ovid PsycINFO 1806 to May Week 3 2017
Date search conducted: 19 May 2017
Strategy:
1 TOLAC*.ti,ab. (3)
2 (trial adj2 labo?r).ti,ab. (21)
3 ((vaginal birth or vaginal delivery) adj2 c?esarean*).ti,ab. (85)
4 VBAC*.ti,ab. (46)
5 or/1-4 [Combined subject headings & text words for VBAC] (113)
6 limit 5 to (english or french) (106)
7 limit 6 to yr="1985-Current" (104)

Database: Conference Proceedings Citation Index – Science (CPSI-S) & Conference Proceedings Citation Index- Social Science & Humanities (CPCI-SSH) --1990-present via Clarivate Analytics
Date search conducted: 2 May 2017
Strategy:
TS=(TOLAC* or "trial of labour" or "trial of labor" or "vaginal birth after caesarean" or "vaginal birth after cesarean" or "vaginal birth following caesarean" or "vaginal birth following cesarean" or VBAC*) Date: 2015-2017 [RF Note: selected 10 from 45]

Database: ProQuest Dissertations & Theses Global
Date search conducted: 2 May 2017
Strategy:
AB,TI(TOLAC* OR (trial NEAR/2 (labor or labour)) OR (("vaginal birth" OR "vaginal delivery") NEAR/2 (caesarean* OR cesarean*)) OR VBAC*)
Date: From January 01 1985 to December 31 2017 ; English only [no French in results set] (90)

Registry: ClinicalTrials.gov
URL: https://clinicaltrials.gov/
Date search conducted: 9 May 2018
Strategy:
Advanced Search >
Other terms: "vaginal birth after cesarean" OR VBAC OR TOLAC OR "trial of labor after cesarean" OR "trial of labour after cesarean" (23)

Appendix 2. Characteristics of included studies

Study;<br>Design;<br>Country, setting;<br>Funding	Population inclusion criteria;<br>Study period	Intervention(s)	Comparator(s)	Proportion of women with successful induction;<br>Proportion of women with successful VBAC	Definition of uterine dehiscence;<br>Proportion of women with uterine dehiscence	Definition of uterine rupture;<br>Proportion of women with uterine rupture
**Aboulfalah (2001)**<br><br>Prospective cohort<br><br>Morocco, setting NR<br><br>Funding NR	Women with indication for induction of labor, single live fetus, vertex presentation, Bishop score of 5 or less, prior cesarean section, and absence of contra-indication to vaginal birth.<br><br>Study period NR	**Induction:**<br>Misoprostol, 50mcg every 6h (intravaginal), max. three doses or until three contractions within 10min<br><br>Bishop's score: mean 2.6 (1.4)<br><br>Co-intervention: oxytocin 22/60 (36.7%)<br><br>n=60 women	**Spontaneous labor:**<br>Spontaneous onset of labor.<br><br>Bishop's score: NR<br><br>Co-intervention: oxytocin NR<br><br>n=300 women	Successful induction within 24h:<br>I: 55/60 (91.7%)<br>C: NR/300<br><br>VBAC:<br>I: 37/60 (61.7%)<br>C: 255/300 (85%), p<0.05	Uterine dehiscence - a defect of uterine wall with intact serosa;<br><br>I: 4/60 (6.7%)<br>C: 4/300 (1.3%), p<0.05	Uterine rupture - complete separation of the uterine wall and serosa, resulting in direct communication between the uterine and the peritoneal cavities;<br><br>I: 1/60 (1.7%)<br>C: 2/300 (0.7%), p>0.05<br><br>note: 2 perinatal deaths occurred in induction group, of which 1 was due to uterine rupture
**Al-Shaikh (2013)**<br><br>Prospective cohort<br><br>Saudi Arabia, University Hospital<br><br>Funding NR	Women with lower segment cesarean section in any previous delivery and admitted for TOLAC.<br><br>April 2010 – March 2011	**Induction:**<br>Bishop's score <6:<br>PGE2, 3mg (vaginal tablet) at 6h interval, max two doses;<br>and/or<br>Foley catheter (intracervical) filled with 30mL distilled water<br><br>Bishop's score ≥6: oxytocin (IV)<br><br>Foley catheter=21 women<br>Oxytocin=19 women<br>PGE2=3 women<br>Combined=19 women<br><br>n=52 women	**Spontaneous labor:**<br>Spontaneous onset of labor.<br><br>n=268 women	Successful induction – NR<br><br>VBAC:<br>I: 33/52 (63.5%)<br>C: 193/268 (72%)	Uterine dehiscence – ND; NR	Uterine rupture – ND;<br><br>I: 1/52 (1.9%)<br>C: 1/268 (0.4%), p=0.32
**Blanco (1992)**	Women with a prior lower	**Induction:**	**Spontaneous labor:**<br>Spontaneous onset of labor.	Successful induction – NR	Uterine scar dehiscence – ND;	Uterine rupture – ND;

Prospective cohort<br><br>USA, Texas Tech University (Lubbock General Hospital)<br><br>Funding NR	segment cesarean section, desire for trial of labor, unfavorable cervix, singleton vertex fetus with reactive non-stress test.<br><br>January 1987 – December 1988	PGE2, 1mg/mL (intracervical gel) during pelvic exam<br><br>n=25 women<br><br>1 gel insertion: 16/25 (64%)<br>2 gel insertion: 4/25 (16%)<br>3 gel insertion: 5/25 (20%)<br><br>Oxytocin for augmentation: 5/25 (20%)	n=56 women<br><br>Oxytocin for augmentation: 9/56 (16.1%)	VBAC:<br>I: 18/25 (72%)<br>C: 46/56 (82.1%),<br>NS	I: 0/25<br>C: 0/56<br><br>Uterine hyperstimulation – ND;<br><br>I: 2/25 (8%)<br>C: 1/56 (1.8%),<br>NS	I: 0/25<br>C: 0/56
**Cieminski (2015)**<br><br>Retrospective cohort<br><br>Poland, Ward of Obstetrics and Gynecology of the Regional Hospital in Chojnice<br><br>Funding NR	Women with one previous low transverse cesarean delivery attempting vaginal birth.<br><br>Study period NR	**Induction/augmentation** with either:<br>Misoprostol, 50mcg administered via insertion in the posterior vaginal fornix. Oxytocin (IV) for facilitated delivery.<br><br>Amniotomy for augmentation =<br><br>n=222 women<br><br>*Oxytocin for augmentation=data NR	**Spontaneous labor:**<br>Women who attempted a spontaneous onset and course of delivery<br><br>n=270 women<br><br>*Oxytocin for augmentation=data NR	Successful induction – NR<br><br>VBAC:<br>I: 168/222 (75.7%)<br>C: 245/270 (90.7%),<br>p<0.001	Uterine dehiscence – ND; NR	Uterine rupture – ND; NR
**Cunha (1999)**<br><br>Prospective cohort<br><br>Mozambique, tertiary health unit with approx. 48,000 deliveries/year to underprivileged and malnourished population; 3,000	Women with previous cesarean section, with indication for induction of labor vs. women who enrolled for a trial of scar<br><br>Study period NR	**Induction:**<br>Misoprostol, 50mcg (intravaginal)<br><br>n=57 women<br><br>Dose repeated once after approximately 18h due to absence of notable uterine activity: 5/57 (8.8%)	**Spontaneous labor:**<br>Women without indication for induction and allowed to deliver spontaneously<br><br>n=57 women	Successful induction – NR<br><br>VBAC:<br>All (spontaneous + assisted):<br>I: 30/57 (52.6%)<br>C: 23/57 (40.4%)<br><br>VD, spontaneous (normal):<br>I: 30/57 (52.6%)<br>C: 21/57 (36.8%);<br>OR 1.9 (95% CI 0.8-4.3)<br><br>VD, assisted (vacuum):<br>I: 0/57<br>C: 2/57 (3.5%)	Uterine dehiscence - ND, NR	Uterine rupture – ND;<br><br>I: 2/57 (3.5%)<br>C: 0/57<br><br>Threatening uterine rupture – ND;<br><br>I: 1/57 (1.8%)<br>C: 2/57 (3.5%);<br>OR 0.5 (95% CI 0.0-9.7)

cesarean deliveries/year<br><br>Non-industry funded				OR 0.0 (95% CI 0-5.3)		
**Flamm (1987)**<br><br>Prospective cohort<br><br>USA, University of California Medical Center & 8 Kaiser Permanente hospitals, serving predominantly indigent and Hispanic population<br><br>Funding NR	Women allowed to labor after a previous cesarean section, without known classical/ low vertical uterine incisions, known previous breech presentation or twin gestation.<br><br>January 1984 – December 1985	**Induction:**<br>Oxytocin (IV) continuous infusion pump to a maximum dose of 20mU/minute<br><br>n=485 women<br><br>Oxytocin administered at cervical dilation:<br>0-2cm: 149/426 (35%)<br>3-4cm: 159/430 (28%)<br>5-10cm: 123/439 (28%)	**No induction:**<br>No oxytocin<br><br>n=1291 women	Successful induction – NR<br><br>VBAC:<br>I: 309/485 (63.7%)<br>C: 1005/1291 (77.8%)<br><br>VD, cervical dilation:	Asymptomatic uterine dehiscence/uterine window – partial-thickness defect with little or no clinical significance;<br><br>I: 5/485 (1.0%)<br>C: 6/1291 (0.5%)<br><br>Uterine dehiscence among women with VD:<br>I: 2/309 (0.6%)<br>C: 2/1005 (0.2%)	True, complete or symptomatic uterine rupture – rupture of the entire uterine wall;<br><br>I: 2/485 (0.4%)<br>C: 1/1291 (0.1%)<br><br>Uterine rupture among women with VD:<br>I: 0/309<br>C: 0/1005
**Flamm (1997)**<br><br>Prospective cohort<br><br>USA, 10 Southern California Kaiser Permanente hospitals<br><br>Funding NR	All pregnant patients with previous cesarean delivery.<br><br>January 1990 - 1992	**Induction:**<br>PGE2, 2-4mg gel (intravaginal) every 4h<br><br>n=453 women<br><br>Oxytocin (induction or augmentation)<br>n=348/453 (76.8%)<br><br>Epidural analgesia (when indicated)<br>n=NR	**No induction:**<br>No PGE2<br><br>n=4569 women<br><br>Oxytocin (induction or augmentation)<br>n=NR<br><br>Epidural analgesia (when indicated)<br>n=NR	Successful induction – NR<br><br>VBAC:<br>All (spontaneous + assisted):<br>I: 233/453 (51.4%)<br>C: 3513/4569 (76.9%), p=0.0001<br><br>VD, spontaneous:<br>I: 196/453 (43.3%)<br>C: NR/4569<br><br>VD, assisted (forceps):<br>I: 8/453 (1.8%)<br>C: NR/4569<br><br>VD, assisted (vacuum):<br>I: 29/453 (6.4%)<br>C: NR/4569	Uterine dehiscence – ND, NR	Uterine rupture – ND;<br><br>I: 6/453 (1.3%)<br>C: 33/4569 (0.7%), p=0.2660<br><br>note: all cases (n=6) in PGE2 group treated with oxytocin after cervical ripening

**Geetha (2012)**<br><br>Prospective cohort<br><br>Oman, Armed Forces Hospital<br><br>No funding	Women with one previous cesarean counselled for vaginal delivery during antenatal period.<br><br>Study period NR	**Induction:**<br>PGE2, 1mg gel repeated at an interval of 6h, max. of three doses, if there was no cervical change<br><br>PGE2, 1 dose: 17/46 (37%)<br>PGE2, 2 dose: 23/46 (50%)<br>PGE2, 3 dose: 6/46 (13%)<br><br>n=46 women<br>(20 women had parity of 4)<br><br>Women with poor uterine action (inadequate contractions) given 2.5 units of oxytocin (infusion) and reassessed after 2h: 10/46 (21.7%)	**Spontaneous labor:**<br>Spontaneous onset of labor.<br><br>n=100 women<br><br>Women with poor uterine action (inadequate contractions) given 2.5units of oxytocin (infusion) and reassessed after 2h=6/100	Successful induction – NR<br><br>VBAC:<br>I: 30/46 (65.2%)<br>C: 79/100 (79%),<br>p=0.116<br><br>VD, women with specified PGE2 doses:<br>1 dose: 12/46 (26.1%)<br>2 dose: 15/46 (32.6%)<br>3 dose: 3/46 (6.5%)	Scar dehiscence – a window in the lower segment with either membranes bulging or parts of the baby visible through;<br><br>I: 0/46<br>C: 0/100	Uterine rupture – an intra-operative finding of fetal parts within the abdominal cavity;<br><br>I: 0/46<br>C: 0/100
**Goldman (1998)**<br><br>Prospective cohort<br><br>Israel, Department of Obstetrics and Gynecology<br><br>Funding NR	Women with a previous cesarean section and without any contraindications to vaginal delivery.<br><br>June 1, 1991 – June 1, 1996	**Induction 1:**<br>Oxytocin (induction/augmentation), 2IU in 1000cc of standard solution (IV)<br><br>n=208 women<br><br>**Induction 2:**<br>PGE2, 1mg (vaginal gel) or 1.5mg (tablet)<br><br>n=146 women	**Spontaneous labor:**<br>Women allowed a trial of spontaneous labor<br><br>n=166 women	Successful induction – NR<br><br>VBAC:<br>I1: 135/208 (64.9%)<br>I2: 105/146 (71.9%)<br>C: 111/166 (66.9%)	Uterine dehiscence – incomplete uterine rupture 'window';<br><br>I1: 1/208 (0.5%)<br>I2: 1/146 (0.7%)<br>C: 0/166	Complete uterine rupture – ND;<br><br>I1: 0/208<br>I2: 0/146<br>C: 0/166<br><br>For women with prior VD:<br>I1 + I2: 9/1558 (0.6%)
**Grobman (2007)**<br><br>Prospective cohort<br><br>USA, 19 medical centers of the National Institute of Child Health and Human Development Maternal-Fetal	Women with one prior low-transverse cesarean and a singleton gestation who underwent a trial of labor at term (>36+6 wks of gestation).<br><br>1999 - 2002	**Induction:**<br>Multiple methods;<br>Amniotomy (no oxytocin or prostaglandin): 84 women<br>Prostaglandin: 140 women<br>Oxytocin without prostaglandin: 2461 women<br>Oxytocin with prostaglandin: 614 women<br><br>n=3259 women	**Spontaneous labor:**<br>Spontaneous onset of labor.<br><br>n=8519 women	Successful induction – NR<br><br>VBAC:<br>I: 2165/3259 (66.4%)<br>C: 6477/8519 (76%)<br><br>VD, women with prior VD:<br>I: 1298/1558 (83.3%)<br>C: 3609/4088 (88.3%);<br>OR 0.66 (85% CI 0.56-0.78), p<0.001	Uterine dehiscence – ND, NR	Uterine rupture – a disruption or tear of the uterine muscle and visceral peritoneum or as a separation of the uterine muscle with extension into the bladder or broad ligament and did not include asymptomatic uterine scar dehiscences;<br><br>I: 35/3259 (1.1%)

Medicine Units Network<br><br>Non-industry funded				VD, women without prior VD:<br>I: 867/1701 (51.0%)<br>C: 2868/4431 (64.7%);<br>OR 0.57 (95% CI 0.51-0.63), p<0.001		C: 54/8519 (0.6%)<br><br>Uterine rupture among women induced with specified agents:<br>No oxytocin or prostaglandin: 0/84<br>Prostaglandin only: 0/140<br>Oxytocin without prostaglandin: 29/2421 (0.9%)<br>Oxytocin with prostaglandin: 6/614 (1.0%)<br><br>Uterine rupture among women with prior VD:<br>I: 9/1558 (0.6%)<br>C: 17/4088 (0.4%);<br>OR 1.39 (95% CI 0.62-3.13), p=0.42<br><br>Uterine rupture among women without prior VD:<br>I: 26/1701 (1.5%)<br>C: 37/4431 (0.8%);<br>OR 1.84 (95% CI 1.11-3.05), p=0.02
**Grubb (1996)**<br><br>RCT<br><br>USA, labor and delivery unit & admitting area<br><br>Funding NR	Term gravidas with one or two unknown uterine scars in early labor who desired a trial of labor, 37-42 wks' gestation, uterine contractions and cervical dilation of <4cm, and singleton vertex presentation.	**Active inpatient management:**<br>Admitted to labor & delivery, received usual care for patients; if uterine contractions led to cervical change, women were allowed a routine trial of labor with oxytocin augmentation when indicated (i.e., persistent contractions without cervical change after 4h)	**Expectant outpatient management:**<br>Allowed to ambulate in admitting area; if no cervical change or spontaneous rupture of membranes within 4h, patient was discharged home with instructions to return for increasing contractions, rupture of membranes, vaginal bleeding or decreased fetal movement; subjects with progressive	Entered labor spontaneously:<br>I: 50/95 (53%)<br>C: 80/93 (86%), p<0.001<br><br>VBAC:<br>All (spontaneous + assisted):<br>I: 80/95 (84%)<br>C: 77/95 (81%)<br><br>VD, spontaneous:<br>I: 63/95 (66%)<br>C: 58/93 (62%), p>0.05 (NS)	Uterine scar disruption – ND;<br>I: 5/95 (5%)<br>C: 0/93, p=0.03<br><br>Note: asymptomatic scar dehiscence, ND (n=4, group assignment NR)	1 vertical scar rupture (ND, group assignment NR) in a woman with two prior cesarean deliveries (one of which was a vertical incision), which led to hysterectomy

	Study period NR	n=95 women<br><br>Oxytocin augmentation according to institutional protocol, initial infusion rate 1mU/minute, allowed increase every 30min if needed, max. dose of 22mU/min.	cervical change admitted & allowed trial of labor with oxytocin augmentation when indicated<br><br>n=95 women<br><br>Oxytocin augmentation as indicated (protocol NR)	VD, assisted (vacuum/forceps):<br>I: 17/95 (18%)<br>C: 19/93 (20%),<br>p>0.05 (NS)		
**Horenstein (1984)**<br><br>Retrospective cohort<br><br>USA, University of Southern California Medical Center<br><br>Non-industry funded	Women with previous cesarean undergoing a trial of labor.<br><br>January 1, 1980 – December 31, 1980	**Induction/augmentation:** Oxytocin when indicated and after consultation with the attending staff, incremental to a max. dose of 22mU/min (IV; Harvard pump).<br><br>n=58 women<br><br>Induction: 12 women<br>Augmentation: 46 women	**No induction/augmentation:** No oxytocin<br><br>n=234 women	Successful induction – NR<br><br>VBAC:<br>I: 31/58 (53.4%)<br>C: 196/234 (83.8%),<br>p<0.005	Uterine dehiscence – ND;<br><br>I: 3/58 (5.2%)<br>C: 3/234 (1.3%)<br>NS<br><br>Uterine atony – ND, among women with vaginal delivery complications:<br>I: 0/31<br>C: 2/197 (1%)	Uterine rupture – ND, NR
**Horenstein (1985)**<br><br>Prospective cohort<br><br>USA, University of Southern California Medical Center<br><br>Funding NR	Women with previous cesarean section undergoing a trial of labor.<br><br>July 1, 1982 – June 30, 1983	**Induction:**<br>Oxytocin; when indicated for obstetric reasons, incremental to a max. dose of 22mU/min (IV; Harvard pump)<br><br>n=289 women<br><br>Induction: 32 women<br>Augmentation: 257 women	**No induction:**<br>No oxytocin<br><br>n=443 women	Successful induction – NR<br><br>VBAC:<br>I: 200/289 (69.2%)<br>C: 395/443 (89.2%),<br>p<0.05<br><br>VD among women whose labor was induced: 23/32 (71.9%)<br>VD among women whose labor was augmented: 77/257 (68.9%)	Uterine dehiscence – ND;<br><br>I: 9/289 (3.1%)<br>C: 6/443 (1.4%)<br><br>Uterine atony – ND, among women with vaginal delivery complications:<br>I: 9/200 (4.5%)<br>C: 8/395 (2.0%)	Uterine rupture – ND, NR
**Kehl (2016)**	Women with singleton	**Induction 1:**	**Induction 2:**	VD within 24h of induction:<br>I1: 37/112 (33%)	Uterine dehiscence – ND, NR	Uterine rupture – ND;

Prospective cohort<br><br>Germany, 4 hospitals in Germany<br><br>No funding	pregnancies, previous cesarean section at term (>= 259 days of gestation), undergoing labor induction at term. Only cases with previous transverse uterotomy were considered.<br><br>January 2012 – December 2013	PGE2, initial dosage of 1mg, followed by 2mg after 6h, if necessary 2mg 24h after initial dose (vaginal gel).<br><br>n=112 women<br><br>Received oxytocin: 45/112 (40.2%)<br>Received epidural analgesia: 40/112 (35.7%)	Double-balloon catheter followed by vaginal PGE2 based on physicians' preference. Balloons filled with 80mL of saline, removed after 12h if catheter did not fall out spontaneously<br><br>n=98 women<br><br>Received oxytocin: 47/98 (48%)<br>Received epidural analgesia: 36/98 (36.7%)<br><br>PGE2 gel given the next morning, if no labor after mechanical ripening (68/98 women)	I2: 25/98 (25.5%)<br><br>VD within 48h of induction:<br>I1: 58/112 (51.8%)<br>I2: 46/98 (46.9%)<br><br>Failed induction (no VBAC within 72h of induction):<br>I1: 6/112 (5.4%)<br>I2: 3/98 (3.1%)<br><br>VBAC:<br>All (spontaneous + assisted):<br>I1: 71/112 (63.4%)<br>I2: 57/98 (58.2%)<br><br>VD, spontaneous (normal):<br>I1: 58/112 (51.8%)<br>47/98 (48%),<br>p=0.736<br><br>VD, assisted (operative):<br>I1: 13/112 (11.6%)<br>I2: 10/98 (10.2%)<br><br>VD, women with prior VD:<br>I1: 38/46 (82.6%)<br>I2: 19/21 (90.5%)<br><br>VD, women without prior VD:<br>I1: 33/66 (0.5%)<br>I2: 38/77 (49.4%)		I: 1/112 (0.9%)<br>I2: 0/98,<br>p=0.499<br><br>Note: case (n=1) of uterine rupture occurred in a women without prior VD
**Lao (1987)**<br><br>Retrospective cohort<br><br>Hong Kong, Princess Margaret Hospital	Women with only one previous lower segment operation and no new or recurrent indications for a repeat operation. Only pregnancies with cephalic presentation at	**Induction:**<br>Oxytocin, dependent on Bishop score.<br>Bishop score >6: forewaters amniotomy performed, oxytocin infusion only added later in contractions did not become established after 1h.	**No induction:**<br>No oxytocin.<br><br>Bishop score <4: Indication for induction was reviewed. If not deferred, cervix was ripened first with vaginal insertion of PGE2 tablet, dose of 3mg.	Successful induction – NR<br><br>VBAC:<br><br>All (spontaneous + assisted):<br>I: 86/102 (84.3%)<br>C: 26/35 (74.3%)<br><br>VD, spontaneous:<br>I: 69/102 (67.6%)	Uterine dehiscence – ND, NR<br><br>Uterine atony – ND;<br>Data not clear, 4 cases among women with postpartum hemorrhage (group assignment NR)	Uterine rupture – ND;<br><br>I: 0/102<br>C: 0/35<br>Note: 1 case of uterine rupture in a patient with a scheduled TOLAC (group assignment NR)

Funding NR	term, obstetric conjugate of more than 10cm and transverse diameter of the inlet more than 11.5cm was accepted.<br><br>1980 - 1983	Bishop score 4-6: amniotomy followed by oxytocin infusion, starting at 4mU/mL, increased stepwise to max of 64mU/mL or until contractions became established. If frank rupture of membranes, oxytocin infusion alone given.<br>Bishop score <4: indication for induction was reviewed. If not deferred, cervix was ripened first with vaginal insertion of PGE2 tablets, dose of 3mg.<br>Analgesia (IM, pethidine) was also given (data NR).<br><br>n=102 women	n=35 women	C: 22/35 (62.9%)<br><br>VD, assisted (instrumental):<br>I: 17/102 (16.7%)<br>C: 4/35 (11.4%)		
**Lelaidier (1994)**<br><br>Prospective double blind placebo controlled trial<br><br>France, setting NR<br><br>Funding NR	Women at term (after 37.5 wks' amenorrhea) who had one previous cesarean delivery with a low transverse uterine incision. All women had a clear clinical indication for induction of labor with unfavorable cervical conditions.<br><br>Maternal age, mean (SD): mifespristone vs. placebo: 33y (4.6) vs. 32y (5.1)<br><br>Study period NR; 6 months	**Induction:**<br>Mifepristone, 200mg (oral) for 2 days.<br><br>n=16 women<br><br>On day 4 (day of planned induction) if:<br><br>Bishop score was <=3, prostaglandin was given (vaginal tablets, 2.5mg).<br>Bishop score >=4, amniotomy, oxytocin infusion and epidural analgesia given.	**Placebo:**<br>Tablet similar in appearance to intervention<br><br>n=16 women<br><br>On day 4 (NR, assumed as per protocol for induction group):<br>Bishop score was <=3, prostaglandin was given (vaginal tablets, 2.5mg).<br>Bishop score >=4, amniotomy, oxytocin infusion and epidural analgesia given.	Successful induction (spontaneous onset of labor):<br>I: 11/16 (68.8%)<br>C: 2/16 (12.5%),<br>p<0.01<br><br>VBAC:<br>All (spontaneous + assisted):<br>I: 11/16 (68.8%)<br>C: 8/16 (50%)<br><br>VD, spontaneous:<br>I: 6/16 (37.5%)<br>C: 4/16 (25%),<br>p>0.05 (NS)<br><br>VD, assisted (operative):<br>I: 5/16 (31.3%)<br>C: 4/16 (25%),<br>p>0.05 (NS)	Uterine scar separation – ND;<br>I: 1/16 (6.3%)<br>C: 1/16 (6.3%)<br><br>Uterine hyperstimulation – ND;<br>I: 0/16<br>C: 0/16	Uterine rupture – ND;<br><br>I: 0/16<br>C: 0/16

**Manish (2016)**<br><br>RCT<br><br>India, large tertiary center; 15,000 deliveries/year<br><br>Non-industry funded	Women with a previous lower segment cesarean section, now with a singleton cephalic presentation after 36 completed weeks, not in labor, with intact membranes, and Bishop's score of <6.<br><br>Maternal age, mean (SD):<br>30mL Foley vs. 80mL Foley: 26.4y (3.2) vs. 26.9y (3.7)<br><br>October 2011 – December 2013	**Induction 1:**<br>30mL Foley catheter, introduced into cervix beyond internal os and bulb inflated with 30mL sterile water, folded and left in vagina for 12h, then removed. Assessment of cervix and amniotomy were done at time of catheter removal or earlier if catheter expelled spontaneously.<br><br>n=77 women<br><br>Oxytocin (induction or augmentation), at rate of 2.5mIU/min (IV), considered if women did not have regular uterine contractions lasting 30sec, every 3min: 62 women	**Induction 2:**<br>80mL Foley catheter, introduced into cervix beyond internal os and bulb inflated with 80mL sterile water, folded and left in vagina for 12h, then removed. Assessment of cervix and amniotomy were done at time of catheter removal or earlier if catheter expelled spontaneously.<br><br>n=77 women<br><br>Oxytocin (induction or augmentation), at rate of 2.5mIU/min (IV), considered if women did not have regular uterine contractions lasting 30sec, every 3min: 50 women	Successful induction - NR<br><br>VBAC:<br>VD within 12h of induction:<br>I: 0/77 (0%)<br>C: 1/77 (1.3%),<br>p>0.99<br><br>VD within 24h of induction:<br>I: 14/77 (18.2%)<br>C: 11/77 (14.3%);<br>RR 1.15 (95% CI 0.78-1.69),<br>p=0.663	Scar dehiscence – full thickness separation of the uterine wall with an intact serosa;<br><br>I: 2/77 (2.6%)<br>C: 7/77 (9.1%);<br>RR 0.28 (95% CI 0.08-1.02),<br>p=0.621	Uterine rupture – full thickness separation of the uterine wall associated with partial or complete extrusion of fetal parts;<br><br>I: 1/77 (1.3%)<br>C: 1/77 (1.3%);<br>RR 1.2 (95% CI 0.26-5.59),<br>p>0.99
**Ogbonmwan (2010)**<br><br>Retrospective cohort<br><br>UK, Wycombe General Hospital<br><br>Funding NR	Women who came for delivery after a primary cesarean section, and agreed to VBAC after counselling.<br><br>Maternal age, mean:<br>Membrane sweep vs. spontaneous labor: 32.2y vs. 32.2y<br><br>January 1, 2001 – December 31, 2006	**Induction:**<br>Membrane sweep<br><br>n=62 women<br><br>Syntocinon augmentation= 2/62 (3.2%)<br>Amniotomy (induction): 28/62 (45.2%)<br>Amniotomy (augmentation): 1/62 (1.6%)<br><br>Analgesia:<br>pethidine: 5 women<br>epidural: 34 women	**Spontaneous labor:**<br>Entered labor spontaneously, without any intervention<br><br>n=79 women<br><br>Syntocinon augmentation: 1/79 (1.3%)<br>Amniotomy (induction): 0/62<br>Amniotomy (augmentation): 47/79 (59.5%)<br><br>Analgesia:<br>pethidine: 8 women<br>epidural: 37 women	Successful induction (spontaneous onset of labor):<br>I: 62/229 (27.1%)<br>C: 79/229 (34.5%)<br><br>VBAC:<br>All (spontaneous + assisted):<br>I: 31/62 (50%)<br>C: 49/79 (62%)<br><br>VD, spontaneous:<br>I: 18/62 (29%)<br>C: 30/79 (38%)<br><br>VD, assisted (instrumental):<br>I: 13/62 (21%)<br>C: 19/79 (24.1%)	Uterine dehiscence – ND; NR	Uterine rupture – ND;<br><br>I: 0/62<br>C: 0/79

		epidural + pethidine: 3 women<br>TENS: 4 women<br>Entonox: 23 women	epidural + pethidine: 4 women<br>TENS: 6 women<br>Entonox: 28 women			
**Palatnik (2015)**<br><br>Retrospective cohort<br><br>USA, Cesarean Registry of the Eunice Kennedy Shriver National Institute of Child Health and Human Development Maternal-Fetal Medicine Units Network<br><br>Funding NR	All women with a history of one cesarean delivery via a low transverse or unknown uterine incision and at least 39 wks of gestation.<br><br>Maternal age, mean (SD):<br>oxytocin:<br>39^0-39^3 wGA: 30.2 (5.4)<br>40^0-40^3 wGA: 29.7 (5.4)<br>41^0-41^3 wGA: 28.7 (5.5)<br>Expectant management:<br>>39^3 wGA: 28.1 (5.7)<br>>40^3 wGA: 27.7 (5.7)<br>>41^3 wGA: 27.4 (5.6)<br><br>Study period NR; Registry data from 1999-2002	**Induction:**<br>Labor induction (unspecified method/agent) during each GA window (39^0-39^3 wk, 40^0-40^3 wk, 41^0-41^3 wk)<br><br>N= 1,631 women	**Expectant management:**<br>Managed expectantly after the GA window of the induction group (>39^3 wk, >40^3 wk, >41^3 wk)<br><br>N= 11,045 women	Successful induction – NR<br><br>VBAC:<br>I: 1,088/1,631 (66.7%)<br>C: 6,787/11,045 (61.4%)<br><br>39^0-39^3 vs. >39^3 wGA:<br>I: 471/638 (73.8%)<br>C: 4640/7565 (61.3%);<br>OR 1.78 (95% CI 1.48-2.13);<br>aOR* 1.31 (95% CI 1.03-1.67)<br><br>40^0-40^3 vs. >40^3 wGA:<br>I: 340/522 (65.1%)<br>C: 1817/2933 (62%);<br>OR 1.15 (95% CI 0.94-1.39);<br>aOR* 1.21 (95% CI 0.93-1.56)<br><br>41^0-41^3 vs. >41^3 wGA:<br>I: 277/471 (58.8%)<br>C: 330/547 (60.3%);<br>OR 0.94 (95% CI 0.73-1.21);<br>aOR* 1.04 (95% CI 0.76-1.43)<br><br>*adjusted for: maternal age, race, recurrent indication for prior cesarean delivery, presence of prior vaginal delivery, presence of prior VBAC	Uterine dehiscence – ND, NR	Uterine rupture – a disruption or tear of the uterine muscle and visceral peritoneum or a separation of the uterine muscle with extension to the bladder or broad ligament;<br><br>39^0-39^3 vs. >39^3 wGA:<br>I: 9/638 (1.4%)<br>C: 40/7565 (0.5%), p<0.05<br><br>40^0-40^3 vs. >40^3 wGA:<br>I: 7/522 (1.3%)<br>C: 17/2933 (0.6%)<br><br>41^0-41^3 vs. >41^3 wGA:<br>I: 6/471 (1.3%)<br>C: 2/547 (0.4%)
**Ramya (2015)**<br><br>RCT<br><br>India, Antenatal outpatient department of	Women with one previous cesarean section with non-recurrent indications, singleton pregnancy and	**Induction:**<br>Membrane sweep; fetal membrane separated from cervix and lower uterine segment as far as possible by sweeping a finger through 360 degrees. If	**No membrane sweep:**<br>Gentle vaginal examination done until the onset of labor.	Successful induction (spontaneous onset of labor):<br>I: 46/75 (61%)<br>C: 48/75 (64%)<br><br>VBAC:<br>I: 13/75 (17.3%)	Scar dehiscence – ND; NR	Uterine rupture – ND;<br><br>I: 0/75<br>C: 0/75

Mahatma Ghandi Medical College and Research Institute<br><br>Funding NR	cephalic presentation, gestational age of 39 wks, intact membrane and candidates willing for VBAC.<br><br>January 2011 – June 2012	cervix was closed, attempt to stretch open or cervical massage was performed. Done at 39 and 40 wks.<br><br>N= 75 women<br><br>*Oxytocin augmentation: 14/75 women (18.7%)	N= 75 women<br><br>*Oxytocin augmentation: 16/75 women (21.3%)	C: 14/75 (18.7%), p=0.532		
**Rayburn (1999)**<br><br>RCT, multicenter<br><br>USA, multiple study centers<br><br>Industry-funded	Term pregnant women who each had one previous low-transverse cesarean and an unfavorable cervix (Bishop score no more than 6), and who was a candidate for vaginal delivery.<br><br>Study period NR	**Induction:**<br>PGE2; 0.5mg in 2.5mL using a catheter into the cervical canal, below internal os. After administration, subjects remained supine for min 15 minutes, electronic FHR and uterine monitoring were continued for at least 2 hours. Return office visits at 40 and 41 weeks when appropriate.<br><br>N= 143 women<br><br>*Oxytocin augmentation or induction: 46/143 (32.2%)	**Expectant management:**<br>Return at 40 and 41 weeks for routine reassessments. Fetal heart rate monitoring done only if clinically indicated at 40 weeks, routinely 41 weeks.<br><br>N= 151 women<br><br>*Oxytocin augmentation or induction: 41/151 (27.2%)	Successful induction - NR<br><br>VBAC (spontaneous + assisted):<br>I: 82/143 (57.3%)<br>C: 83/151 (55.0%), p=0.68<br><br>VD, spontaneous:<br>I: 70/143 (49.0%)<br>C: 74/151 (49.0%)<br><br>VD, assisted (instrumental):<br>I: 12/143 (8.4%)<br>C: 9/151 (6.0%)	Uterine dehiscence – ND; NR<br><br>Uterine hyperstimulation – ND;<br>I: 1/143 (0.7%)<br>C: 0/151	Uterine rupture – ND;<br><br>I: 0/143<br>C: 0/151
**Sakala (1990)**<br><br>Retrospective cohort<br><br>USA, Loma Linda University Medical Center<br><br>Funding NR	Women with at least one previous low segment transverse cesarean delivery who requested a trial of labor.<br><br>October 1984 – April 1986	**Induction/augmentation:**<br>Oxytocin for induction (without uterine contractions) & augmentation (with uterine contractions). Infusion pump starting at 0.5mU/minute, advancing every 15 minutes as needed to max of 24mU/min. External tococardiography used with intact membranes, direct scalp electrode placed with ruptured membranes.	**No induction/augmentation:**<br>No oxytocin.	Successful induction - NR<br><br>VBAC:<br>I: 50/73 (68.5%)<br>C: 146/164 (89%)<br><br>VD, assisted (operative)*:<br>I: 12/73 (16.4%)<br>C: 29/164 (17.7%)<br><br>VD among women whose labor was induced:<br>28/48 (58.3%)<br>VD among women whose labor was augmented:	Uterine scar dehiscence – asymptomatic, silent separation of the previous uterine incision unassociated with perinatal morbidity;<br><br>I: 3/73 (4.1%)<br>C: 1/164 (0.6%)	Overt uterine rupture – symptomatic separation of the previous uterine incision associated with perinatal morbidity;<br><br>I: 0/73<br>C: 0/164

		Intrauterine pressure catheter placed whenever possible with oxytocin administration after membranes ruptured.<br><br>N= 73 women<br><br>Induction: 48/73 (65.8%)<br>Augmentation: 25/73 (34.2%)	N= 164 women	22/25 (88%)<br><br>*study did not report clearly number of operative vaginal deliveries as distinct from (or included in total) number of all vaginal deliveries		
**Sakala (1990)**<br><br>Retrospective cohort<br><br>USA, Loma Linda University Medical Center<br><br>Funding NR	Women with at least one previous low segment transverse cesarean delivery who requested a trial of labor.<br><br>October 1984 – April 1986	**Epidural analgesia:**<br>Epidural catheter preloaded with 750mL of Ringer's lactate, placed at L2-L4 level. Continuous infusion or intermittent bolus given using 0.125% or 0.25% bupivacaine, titrated through first stage of labor for patient comfort. Some women also received oxytocin.<br><br>N= 87 women<br><br>Oxytocin: 40/87 (46%):<br>Oxytocin induction: 35/87 (40.2%);<br>Oxytocin augmentation: 20/87 (23%)	**No epidural analgesia:**<br>Did not receive epidural analgesia. Some women also received oxytocin.<br><br>N=150 women<br><br>Oxytocin: 31/150 (20.7%):<br>Oxytocin induction: 10/150 (6.7%);<br>Oxytocin augmentation: 22/150 (14.7%)	Successful induction – NR<br><br>VBAC (spontaneous + assisted):<br>I: 77/87 (88.5%)<br>C: 125/150 (83.3%)<br><br>VD, spontaneous:<br>I: 48/77 (62.3%)<br>C: 96/125 (76.8%)<br><br>VD, assisted (operative):<br>I: 28/77 (36.4%)<br>C: 29/125 (23.2%), $p<0.05$<br><br>VD, oxytocin for induction or augmentation*:<br>I: 33/77 (42.9%)<br>C: 17/125 (13.6%)<br><br>VD, oxytocin for induction:<br>I: 29/77 (37.7%)<br>C: 8/125 (6.4%)<br><br>VD, oxytocin for augmentation:<br>I: 19/77 (24.7%)<br>C: 9/125 (7.2%)	Scar dehiscence – ND;<br><br>I: 4/87 (4.6%)<br>C: 1/150 (0.7%)	Overt uterine rupture – ND;<br><br>I: 0/87<br>C: 0/150

				*some women received oxytocin for both induction and augmentation		
**Shah (2017)**<br><br>Retrospective cohort<br><br>USA, Ben Taub Hospital<br><br>Funding NR	Women with live, cephalic, singleton gestations of 24 wks of gestation or greater, with at least one prior cesarean delivery who underwent induction of labor with an unfavorable cervix (Bishop score ≤3) and received a Cook balloon for mechanical cervical ripening or oxytocin for induction.<br><br>July 1, 2009 – December 31, 2013	**Induction 1:**<br>Oxytocin; initiated at 1 or 2mU/min and increased by 1 or 2 mU/min every 30min to a max. of 40mU/min.<br><br>N= 150 women<br><br>Epidural analgesia: 138/150 (92.0%)	**Induction 2:**<br>Oxytocin + Cook balloon; inflated with 80/80cc of normal saline, kept in place for 12h until spontaneously expulsed.<br><br>N= 64 women<br><br>Epidural analgesia: 62/64 (96.9%)	Successful induction – hours from start of induction until vaginal delivery;<br>I: mean 15.7h (6.4)<br>C: mean 23.8h (8.1)<br><br>VBAC*:<br>I: 106/150 (70.7%)<br>C: 32/64 (50.0%),<br>p=0.004<br><br>*includes operative vaginal deliveries by forceps or vacuum (number NR)	Asymptomatic uterine scar dehiscence – separation of muscle with intact overlying peritoneum (excluded from study)	Uterine rupture – full thickness separation of all layers of the uterine wall;<br><br>I: 2/150 (1.3%)<br>C: 0/64 (0.0%),<br>NS
**Shatz (2013)**<br><br>Retrospective cohort<br><br>Israel, Soroka University Medical Center<br><br>Funding NR	Women with a singleton fetus in a vertex position who attempted labor after a prior low transverse cesarean section.<br><br>1988 - 2005	**Induction:**<br>Surgical, Foley catheter, oxytocin, prostaglandins.<br><br>N= 1,576 women<br><br>Single method: 1,259/1576 (79.9%);<br>Multiple methods: 314/1576 (19.9%):<br>Two methods: 277/1576 (17.6%)<br>Three or more methods: 39/1576 (2.5%)<br>Surgical: 575/1576 (36.5%)	**Spontaneous labor:**<br>Spontaneous trial of labor/delivery.<br><br>N= 4,263 women	Successful induction – ND; NR<br><br>VBAC:<br>I: 1,062/1,576 (67.4%)<br>C: 3,111/4,263 (73.0%),<br>p<0.001<br><br>VD, single vs. multiple induction methods:<br>920/1,259 (73.0%) vs. 142/314 (45.2%),<br>p<0.001	Uterine scar dehiscence – opening of the previous cesarean scar with intact visceral peritoneum and no direct communication between the uterine and abdominal cavities;<br><br>I: 13/1576 (0.8%)<br>C: 36/4263 (0.8%)<br><br>Uterine dehiscence among women with VD:<br>I: 2/1062 (0.2%)<br>C: 2/3111 (0.1%)	Uterine rupture – complete tear of the uterine wall, including the visceral peritoneum, with establishment of a direct communication between the uterine and abdominal cavities;<br><br>I: 6/1,576 (0.4%)<br>C: 9/4,263 (0.2%)

		Oxytocin: 254/1576 (16.1%)<br>Prostaglandins: 54/1576 (3.4%)<br>Foley catheter: 375/1576 (23.8%)			Uterine dehiscence, single vs. multiple induction methods:<br>7/1259 (0.6%) vs. 6/314 (1.9%), p=0.029	
**Sims (2001)**<br><br>Retrospective cohort<br><br>USA, Perinatal Network database<br><br>Funding NR	Women with a previous cesarean delivery who are candidates for VBAC.<br><br>September 1997 – December 1999	**Induction:**<br>In the form of oxytocin only; misoprostol, 25 or 50mcg (intravaginal) every 4h, max. total of 3 doses, augmented with oxytocin); dinoprostone (cervidil inserted into vagina for 12h, augmented with oxytocin)<br><br>N= 57 women<br><br>Oxytocin augmentation: NR	**Spontaneous labor:**<br>Spontaneous trial of labor.<br><br>N= 179 women<br><br>Oxytocin augmentation: NR	Successful induction – NR<br><br>VBAC:<br>I: 33/57 (57.9%)<br>C: 138/179 (77.1%);<br>OR 2.45 (95% CI 1.24-4.82), p=0.008<br><br>VD among women induced with oxytocin:<br>I: NR (87%)<br>C: NR (64.5%);<br>OR -3.67 (95% CI -1.65—8.28), p=0.0008	Asymptomatic dehiscence – ND (combined with uterine rupture); NR	Symptomatic rupture – ND (uterine scar separation=asymptomatic dehiscence + symptomatic rupture); NR
**Taylor (1993)**<br><br>RCT<br><br>UK, setting NR<br><br>Funding NR	Women with previous pregnancy delivered by lower segment cesarean section, presenting at least 37 wks' gestation, singleton pregnancy, cephalic presentation and a modified Bishop score <9.<br><br>Study period NR	**Induction 1:**<br>Low amniotomy and immediate IV oxytocin titration; oxytocin (augmentation) if no labor after 6h.<br><br>N= 21 women<br><br>Analgesia:<br>Entonox: 4/21 (19%)<br>Pethidine: 5/21 (23.8)<br>Epidural: 12/21 (57.1%)	**Induction 2:**<br>Vaginal administration of PGE2 (2.5mg) followed by low amniotomy 3h later; oxytocin (augmentation) if no labor after 6h.<br><br>N= 21 women<br><br>Analgesia:<br>Entonox: 4/21 (19%)<br>Pethidine: 0/21<br>Epidural: 17/21 (81%)	Induction-to-delivery interval:<br>I: mean 8.9h (2.4)<br>C: mean 10.8h (4.2)<br><br>VBAC (spontaneous + assisted):<br>I: 15/21 (71.4%)<br>C: 17/21 (81.0%)<br><br>VD, spontaneous:<br>I: 11/21 (52.4%)<br>C: 12/21 (57.1%)<br><br>VD, assisted (instrumental):<br>I: 4/21 (19.0%)<br>C: 5/21 (23.8%)	Uterine dehiscence – ND; NR	Uterine rupture – rupture of uterus/scar;<br><br>I: 0/21 (0%)<br>C: 1/21 (4.8%), occurred after oxytocin augmentation
**Tussupkaliyer (2016)**<br><br>Prospective cohort	Pregnant women with a previous cesarean section in their medical history.	**Induction 1:**<br>Bishop score 6-8; misoprost<br><br>**Induction 2:**<br>Bishop >8 and lack of regular contractions; amniotomy	**Induction 3:**<br>Disrupted fetal membrane<br><br>**Spontaneous labor:**<br>Spontaneous onset of labor.	Successful induction – NR<br><br>VBAC:<br>I: NR/83 (70.0%), RR 0.86<br>I2: NR/39 (63.5%), RR 0.76	Uterine dehiscence – ND; NR	Uterine rupture – disruption of previous scar, occurred asymptomatically; diagnosed during

Kazakhstan, West-Kazakhstan Marat Ospanov State Medical University<br><br>Funding NR	Study period NR	ol (25mcg every 6h, max. 100mcg).<br><br>N= 89 women	with vulsellum jaws.<br><br>N= 62 women	; Oxytocin IV infusion<br><br>N= 95 women	N= 96 women	C: NR/88 (62.0%), RR 0.83		postpartum period due to clinical signs of internal hemorrhage and small pelvis and abdomen ultrasound exam;<br><br>I1: 0/89<br>I2: 0/62<br>I3: 0/95<br>C: 3/96 (3.1%)
**Yogev (2004)**<br><br>Retrospective cohort<br><br>Israel, setting NR<br><br>Funding NR	Women with a history of one low-transverse cesarean delivery, singleton pregnancy, cephalic presentation, gravidity <5 and sonographically estimated fetal weight of <4000g.<br><br>January 2002 – December 2002	**Induction:**<br>PGE2, 3mg (intravaginal tablet) applied to posterior cervical fornix. If no change after single dose, additional tablets were given at 6-8h intervals. Bishop score >=7 transferred to delivery room and labor was further augmented with oxytocin or amniotomy, followed by intrauterine pressure catheter.<br><br>N= 97 women<br><br>Oxytocin augmentation: 24/97 (24.7%)		**Spontaneous labor:**<br>Spontaneous onset of labor. Bishop score >=7 transferred to delivery room and labor was further augmented with oxytocin or amniotomy, followed by intrauterine pressure catheter.<br><br>N= 931 women<br><br>Oxytocin augmentation: 241/931 (25.8%)		Successful induction – NR<br><br>VBAC (spontaneous + assisted):<br>I: 62/97 (63.9%)<br>C: 584/931 (62.7%)<br><br>VD, spontaneous:<br>I: 58/97 (59.8%)<br>C: 524/931 (56.3%)<br><br>VD, assisted (operative):<br>I: 4/97 (4.1%)<br>C: 60/931 (6.4%);<br>OR 0.62 (95% CI 0.22-1.75), p=0.36	Uterine dehiscence – ND; NR	Symptomatic uterine rupture – complete disruption of the prior uterine scar in association with at least one of the following symptoms or signs: laparotomy for hemorrhage or hemoperitoneum, excessive injury to the bladder or extrusion into the peritoneal cavity of any portion of the fetal-placental unit, cesarean delivery for nonreassuring fetal heart rate tracing, or suspected rupture as evidenced by the acute onset of incisional pain;<br><br>I: 0/97 (0.0%)<br>C: 4/931 (0.42%), NS

C: comparator(s); CI: confidence interval; GA: gestational age; h: hour(s); I: intervention(s); IM: intramuscular; IV: intravenous; mg: milligram(s); max.: maximum; min: minute(s); n: number; NR: not reported; OR: odds ratio; p: p value; PGE1: prostaglandin E1; PGE2: prostaglandin E2; RCT: randomized controlled trial; RR: relative risk; sec: second(s); TOLAC: trial of labor after cesarean; UK: United Kingdom; wk(s): week(s); VD: vaginal delivery

Appendix 3. Methodological quality of included studies

**MMAT* criteria**	**Author's judgment**	**Support for judgment**
**Aboulfalah, 2001 (Prospective cohort)**		
Are there clear qualitative and quantitative research questions (or objectives), or a clear mixed methods question (or objective)?	Yes	To assess the safety and efficiency of intravaginal misoprostol in women with a prior cesarean.
Do the collected data allow address the research question (objective)? E.g. consider whether the follow-up period is long enough for the outcome to occur (for longitudinal studies or study components).	Yes	Reported delivery outcomes.
3.1 Are participants (organizations) recruited in a way that minimizes selection bias?	Can't tell	Not reported, control group "drawn casually".
3.2 Are measurements appropriate (clear origin, or validity known, or standard instrument; and absence of contamination between groups when appropriate) regarding the exposure/intervention and outcomes?	Can't tell	22/60 (36.7%) women also received oxytocin in addition to misoprostol; unclear whether those in spontaneous labor group also received oxytocin.
3.3 In the groups being compared (exposed vs non-exposed; with intervention vs without; cases vs controls), are the participants comparable, or do researchers take into account (control for) the difference between these groups?	Yes	No significant differences between the groups.
3.4 Are there complete outcome data (80% or above), and, when applicable, an acceptable response rate (60% or above), or an acceptable follow-up rate for cohort studies (depending on the duration of follow-up)?	Yes	Delivery outcomes reported for both groups.
Overall quality score	** 50%	
**Al-Shaikh, 2013 (Prospective cohort)**		
Are there clear qualitative and quantitative research questions (or objectives), or a clear mixed methods question (or objective)?	Yes	To determine the success rate of VBAC and its outcome when labor was induced compared to spontaneous labor.
Do the collected data allow address the research question (objective)? E.g. consider whether the follow-up period is long enough for the outcome to occur (for longitudinal studies or study components).	Yes	Reported delivery outcomes.
3.1 Are participants (organizations) recruited in a way that minimizes selection bias?	Yes	All women who had LCSC in any previous delivery at hospital.
3.2 Are measurements appropriate (clear origin, or validity known, or standard instrument; and absence of contamination between groups when appropriate) regarding the exposure/intervention and outcomes?	Yes	No co-interventions noted, VBAC and delivery outcomes reported.
3.3 In the groups being compared (exposed vs non-exposed; with intervention vs without; cases vs controls), are the participants comparable, or do researchers take into account (control for) the difference between these groups?	Yes	No significant differences between the groups.
3.4 Are there complete outcome data (80% or above), and, when applicable, an acceptable	Yes	Delivery outcomes reported for both groups.

response rate (60% or above), or an acceptable follow-up rate for cohort studies (depending on the duration of follow-up)?		
Overall quality score	**** 100%	
**Blanco, 1992 (Prospective cohort)**		
Are there clear qualitative and quantitative research questions (or objectives), or a clear mixed methods question (or objective)?	Yes	To determine the safety and efficacy of PGE2 gel for induction of labor or ripening of the cervix in patients with a prior LCTCS for a trial of labor, an unfavorable cervix, and a medical indication for delivery.
Do the collected data allow address the research question (objective)? E.g. consider whether the follow-up period is long enough for the outcome to occur (for longitudinal studies or study components).	Yes	Reported delivery outcomes.
3.1 Are participants (organizations) recruited in a way that minimizes selection bias?	Can't tell	No clear description.
3.2 Are measurements appropriate (clear origin, or validity known, or standard instrument; and absence of contamination between groups when appropriate) regarding the exposure/intervention and outcomes?	Yes	Women in both groups also received oxytocin.
3.3 In the groups being compared (exposed vs non-exposed; with intervention vs without; cases vs controls), are the participants comparable, or do researchers take into account (control for) the difference between these groups?	Yes	No significant differences between the two groups.
3.4 Are there complete outcome data (80% or above), and, when applicable, an acceptable response rate (60% or above), or an acceptable follow-up rate for cohort studies (depending on the duration of follow-up)?	Yes	Delivery outcomes reported for both groups, included in the analysis.
Overall quality score	*** 75%	
**Cieminski, 2015 (Retrospective cohort)**		
Are there clear qualitative and quantitative research questions (or objectives), or a clear mixed methods question (or objective)?	Yes	To determine the success and safety of active management of labor including induction and augmentation in women with a prior cesarean section.
Do the collected data allow address the research question (objective)? E.g. consider whether the follow-up period is long enough for the outcome to occur (for longitudinal studies or study components).	Yes	Reported delivery outcomes.
3.1 Are participants (organizations) recruited in a way that minimizes selection bias?	Yes	Patients with a history of a single low transverse incision cesarean section who gave birth at one hospital.
3.2 Are measurements appropriate (clear origin, or validity known, or standard instrument; and absence of contamination between groups when appropriate) regarding the exposure/intervention and outcomes?	No	Induction/augmentation group separated out (oxytocin vs. PGE1 vs. oxytocin + PGE1); Some women received amniotomy in addition to pharmacological interventions, but no data provided.
3.3 In the groups being compared (exposed vs non-exposed; with intervention vs without; cases vs controls), are the participants comparable, or do	No	No reports on whether the group were comparable.

researchers take into account (control for) the difference between these groups?		
3.4 Are there complete outcome data (80% or above), and, when applicable, an acceptable response rate (60% or above), or an acceptable follow-up rate for cohort studies (depending on the duration of follow-up)?	Yes	Delivery outcomes reported for groups, all included in analysis.
Overall quality score	** 50%	
**Cunha, 1999 (Prospective cohort)**		
Are there clear qualitative and quantitative research questions (or objectives), or a clear mixed methods question (or objective)?	Yes	To elucidate whether vaginal misoprostol for labor induction in women with previous cesarean delivery would be a worthwhile alternative management in terms of pregnancy outcome for the mother and the newborn.
Do the collected data allow address the research question (objective)? E.g. consider whether the follow-up period is long enough for the outcome to occur (for longitudinal studies or study components).	Yes	Reported delivery and neonatal outcomes.
3.1 Are participants (organizations) recruited in a way that minimizes selection bias?	Can't tell	Not clear on how selection occurred.
3.2 Are measurements appropriate (clear origin, or validity known, or standard instrument; and absence of contamination between groups when appropriate) regarding the exposure/intervention and outcomes?	Yes	No co-interventions reported for either group.
3.3 In the groups being compared (exposed vs non-exposed; with intervention vs without; cases vs controls), are the participants comparable, or do researchers take into account (control for) the difference between these groups?	Can't tell	No baseline demographics.
3.4 Are there complete outcome data (80% or above), and, when applicable, an acceptable response rate (60% or above), or an acceptable follow-up rate for cohort studies (depending on the duration of follow-up)?	Yes	Delivery outcomes reported for both groups.
Overall quality score	** 50%	
**Flamm, 1987 (Prospective cohort)**		
Are there clear qualitative and quantitative research questions (or objectives), or a clear mixed methods question (or objective)?	Yes	To evaluate the outcome of oxytocin administration in patients with previous cesarean sections who undergo a trial of labor.
Do the collected data allow address the research question (objective)? E.g. consider whether the follow-up period is long enough for the outcome to occur (for longitudinal studies or study components).	Yes	Reported labor and delivery, and maternal and perinatal outcomes.
3.1 Are participants (organizations) recruited in a way that minimizes selection bias?	Can't tell	Doesn't specify if all women were included or if there was a selection process.
3.2 Are measurements appropriate (clear origin, or validity known, or standard instrument; and absence of contamination between groups when	Yes	No co-interventions reported in either group.

appropriate) regarding the exposure/intervention and outcomes?		
3.3 In the groups being compared (exposed vs non-exposed; with intervention vs without; cases vs controls), are the participants comparable, or do researchers take into account (control for) the difference between these groups?	Can't tell	No description of group characteristics.
3.4 Are there complete outcome data (80% or above), and, when applicable, an acceptable response rate (60% or above), or an acceptable follow-up rate for cohort studies (depending on the duration of follow-up)?	Yes	Delivery outcomes reported for both groups.
Overall quality score	** 50%	
**Flamm, 1997**<br>**(Prospective cohort)**		
Are there clear qualitative and quantitative research questions (or objectives), or a clear mixed methods question (or objective)?	Yes	To evaluate the maternal and fetal outcomes of a large cohort of women treated with PGE2 gel for cervical ripening prior to trial of labor after previous cesarean delivery.
Do the collected data allow address the research question (objective)? E.g. consider whether the follow-up period is long enough for the outcome to occur (for longitudinal studies or study components).	Yes	Reported labor and delivery outcomes, including uterine rupture.
3.1 Are participants (organizations) recruited in a way that minimizes selection bias?	Yes	All pregnant women were included.
3.2 Are measurements appropriate (clear origin, or validity known, or standard instrument; and absence of contamination between groups when appropriate) regarding the exposure/intervention and outcomes?	Can't tell	Oxytocin also given but only reported for PGE2 group (77%); epidural analgesia reported but rates are NR.
3.3 In the groups being compared (exposed vs non-exposed; with intervention vs without; cases vs controls), are the participants comparable, or do researchers take into account (control for) the difference between these groups?	Can't tell	Did not report whether the groups were comparable.
3.4 Are there complete outcome data (80% or above), and, when applicable, an acceptable response rate (60% or above), or an acceptable follow-up rate for cohort studies (depending on the duration of follow-up)?	Yes	Delivery outcomes reported for both groups.
Overall quality score	** 50%	
**Geetha, 2012**<br>**(Prospective cohort)**		
Are there clear qualitative and quantitative research questions (or objectives), or a clear mixed methods question (or objective)?	Yes	To study the outcome of induction of labor with PGE2 vaginal gel in those with one previous cesarean section.
Do the collected data allow address the research question (objective)? E.g. consider whether the follow-up period is long enough for the outcome to occur (for longitudinal studies or study components).	Yes	Reported labor and delivery, and neonatal outcomes.
3.1 Are participants (organizations) recruited in a way that minimizes selection bias?	Can't tell	Unclear if consecutive patients, study data not clear, unclear on how selection occurred.
3.2 Are measurements appropriate (clear origin, or validity known, or standard instrument; and	Can't tell	Reports women with poor uterine action also given oxytocin (rates NR),

absence of contamination between groups when appropriate) regarding the exposure/intervention and outcomes?		unclear if no PGE2 comparator group also received oxytocin.
3.3 In the groups being compared (exposed vs non-exposed; with intervention vs without; cases vs controls), are the participants comparable, or do researchers take into account (control for) the difference between these groups?	Yes	No difference in the age group or parity.
3.4 Are there complete outcome data (80% or above), and, when applicable, an acceptable response rate (60% or above), or an acceptable follow-up rate for cohort studies (depending on the duration of follow-up)?	Yes	Delivery outcomes reported for both groups.
Overall quality score	** 50%	
**Goldman, 1998**<br>**(Prospective cohort)**		
Are there clear qualitative and quantitative research questions (or objectives), or a clear mixed methods question (or objective)?	Yes	To compare obstetric outcomes of induction of labor with oxytocin or PGE2, with spontaneous labor, in patients with one previous cesarean section who underwent a trial of labor.
Do the collected data allow address the research question (objective)? E.g. consider whether the follow-up period is long enough for the outcome to occur (for longitudinal studies or study components).	Yes	Reported delivery and obstetric outcomes.
3.1 Are participants (organizations) recruited in a way that minimizes selection bias?	Yes	All women with previous cesarean section undergoing trial of labor in department.
3.2 Are measurements appropriate (clear origin, or validity known, or standard instrument; and absence of contamination between groups when appropriate) regarding the exposure/intervention and outcomes?	Yes	No co-interventions reported in any groups.
3.3 In the groups being compared (exposed vs non-exposed; with intervention vs without; cases vs controls), are the participants comparable, or do researchers take into account (control for) the difference between these groups?	Yes	Patients of similar age, parity and indication for previous section.
3.4 Are there complete outcome data (80% or above), and, when applicable, an acceptable response rate (60% or above), or an acceptable follow-up rate for cohort studies (depending on the duration of follow-up)?	Yes	Delivery outcomes reported for both groups.
Overall quality score	**** 100%	
**Grobman, 2007**<br>**(Prospective cohort)**		
Are there clear qualitative and quantitative research questions (or objectives), or a clear mixed methods question (or objective)?	Yes	To compare pregnancy outcomes in women with one prior low transverse cesarean delivery after induction of labor with pregnancy outcomes after spontaneous labor.
Do the collected data allow address the research question (objective)? E.g. consider whether the follow-up period is long enough for the outcome to occur (for longitudinal studies or study components).	Yes	Reported delivery, obstetric and neonatal outcomes.

3.1 Are participants (organizations) recruited in a way that minimizes selection bias?	Yes	All women with prior cesarean delivery from 19 academic medical centers (part of a maternal-fetal network).
3.2 Are measurements appropriate (clear origin, or validity known, or standard instrument; and absence of contamination between groups when appropriate) regarding the exposure/intervention and outcomes?	Can't tell	Not clear on women who did not receive any oxytocin or prostaglandin; may have received artificial rupture of membranes but unclear.
3.3 In the groups being compared (exposed vs non-exposed; with intervention vs without; cases vs controls), are the participants comparable, or do researchers take into account (control for) the difference between these groups?	Can't tell	Demographics stratified by type of labor and history of vaginal delivery; women with induction more likely to be older, white, married and have higher BMI. No mention of controlling for differences.
3.4 Are there complete outcome data (80% or above), and, when applicable, an acceptable response rate (60% or above), or an acceptable follow-up rate for cohort studies (depending on the duration of follow-up)?	Yes	Delivery outcomes reported for both groups.
Overall quality score	** 50%	
**Grubb, 1996**<br>**(RCT)**		
Are there clear qualitative and quantitative research questions (or objectives), or a clear mixed methods question (or objective)?	Yes	To determine if expectant outpatient management would decrease risk of protracted labor and cesarean delivery compared with standard active inpatient management.
Do the collected data allow address the research question (objective)? E.g. consider whether the follow-up period is long enough for the outcome to occur (for longitudinal studies or study components).	Yes	Reported delivery, obstetric and neonatal outcomes.
2.1 Is there a clear description of the randomization (or an appropriate sequence generation)?	Yes	Random permuted block technique with blocks of six to group assignment.
2.2 Is there a clear description of the allocation concealment (or blinding when applicable)?	Can't tell	Group assignment from one of the investigators by phone.
2.3 Are there complete outcome data (80% or above)?	Yes	9 (5%) women with incomplete data. 92-95% outcome data.
2.4 Is there low withdrawal/drop-out (below 20%)?	Yes	9 (5%) women lost to follow-up.
Overall quality score	*** 75%	
**Horenstein, 1984**<br>**(Retrospective cohort)**		
Are there clear qualitative and quantitative research questions (or objectives), or a clear mixed methods question (or objective)?	Yes	To investigate risks associated with oxytocin usage in patients with prior cesarean section undergoing a trial of labor.
Do the collected data allow address the research question (objective)? E.g. consider whether the follow-up period is long enough for the outcome to occur (for longitudinal studies or study components).	Yes	Reported delivery, obstetric and neonatal outcomes.
3.1 Are participants (organizations) recruited in a way that minimizes selection bias?	Yes	All women from medical center who underwent trial of labor.
3.2 Are measurements appropriate (clear origin, or validity known, or standard instrument; and absence of contamination between groups when appropriate) regarding the exposure/intervention and outcomes?	Yes	No co-interventions reported for either groups.

3.3 In the groups being compared (exposed vs non-exposed; with intervention vs without; cases vs controls), are the participants comparable, or do researchers take into account (control for) the difference between these groups?	Yes	Groups were comparable regarding number of prior uterine incisions, cephalopelvic disproportion, failure to progress, and number of prior vaginal deliveries.
3.4 Are there complete outcome data (80% or above), and, when applicable, an acceptable response rate (60% or above), or an acceptable follow-up rate for cohort studies (depending on the duration of follow-up)?	Yes	292/308 (95%) patients are included in the analysis. Delivery outcomes, complications and neonatal outcomes reported.
Overall quality score	**** 100%	
**Horenstein, 1985<br>(Prospective cohort)**		
Are there clear qualitative and quantitative research questions (or objectives), or a clear mixed methods question (or objective)?	Yes	To investigate the role of oxytocin in women with prior cesarean who undergo a trial of labor.
Do the collected data allow address the research question (objective)? E.g. consider whether the follow-up period is long enough for the outcome to occur (for longitudinal studies or study components).	Yes	Reported delivery, obstetric and neonatal outcomes.
3.1 Are participants (organizations) recruited in a way that minimizes selection bias?	Yes	All women in medical center with one prior cesarean.
3.2 Are measurements appropriate (clear origin, or validity known, or standard instrument; and absence of contamination between groups when appropriate) regarding the exposure/intervention and outcomes?	Yes	No co-interventions reported for either groups. VBAC and delivery outcomes measured.
3.3 In the groups being compared (exposed vs non-exposed; with intervention vs without; cases vs controls), are the participants comparable, or do researchers take into account (control for) the difference between these groups?	Can’t tell	No description of group characteristics. Did not report whether groups were comparable.
3.4 Are there complete outcome data (80% or above), and, when applicable, an acceptable response rate (60% or above), or an acceptable follow-up rate for cohort studies (depending on the duration of follow-up)?	Yes	Delivery outcomes, complication and neonatal outcomes reported for both groups.
Overall quality score	*** 75%	
**Kehl, 2016<br>(Prospective cohort)**		
Are there clear qualitative and quantitative research questions (or objectives), or a clear mixed methods question (or objective)?	Yes	To evaluate the efficacy of inducing labor using a double-balloon catheter and vaginal PGE2 sequentially, in comparison with vaginal PGE2 alone after a previous cesarean section.
Do the collected data allow address the research question (objective)? E.g. consider whether the follow-up period is long enough for the outcome to occur (for longitudinal studies or study components).	Yes	Reported delivery, obstetric and neonatal outcomes.
3.1 Are participants (organizations) recruited in a way that minimizes selection bias?	Yes	All women from four German hospitals with previous cesarean section, transverse incision were included.
3.2 Are measurements appropriate (clear origin, or validity known, or standard instrument; and absence of contamination between groups when	Can’t tell	PGE2 group also received oxytocin (40%) and analgesia (36%); PGE2 and balloon catheter group also received

appropriate) regarding the exposure/intervention and outcomes?		oxytocin (48%) and analgesia (37%); no significant differences between groups.
3.3 In the groups being compared (exposed vs non-exposed; with intervention vs without; cases vs controls), are the participants comparable, or do researchers take into account (control for) the difference between these groups?	Yes	Table 1 compares baseline demographics, reports higher proportion with >=1 previous vaginal delivery in the PGE2 groups (p=0.002). Outcome parameters compared in relation to parity (Table 4).
3.4 Are there complete outcome data (80% or above), and, when applicable, an acceptable response rate (60% or above), or an acceptable follow-up rate for cohort studies (depending on the duration of follow-up)?	Yes	Delivery outcomes, complications and neonatal outcomes reported for both groups.
Overall quality score	*** 75%	
**Lao, 1987**<br>**(Retrospective cohort)**		
Are there clear qualitative and quantitative research questions (or objectives), or a clear mixed methods question (or objective)?	Yes	To evaluate the safety of induction for a trial of scar and the rate of repeat cesarean in patients with previous cesarean.
Do the collected data allow address the research question (objective)? E.g. consider whether the follow-up period is long enough for the outcome to occur (for longitudinal studies or study components).	Yes	Reported delivery, obstetric and neonatal outcomes.
3.1 Are participants (organizations) recruited in a way that minimizes selection bias?	Yes	All women from one hospital with previous lower segment cesarean section.
3.2 Are measurements appropriate (clear origin, or validity known, or standard instrument; and absence of contamination between groups when appropriate) regarding the exposure/intervention and outcomes?	Yes	No co-interventions reported for either groups.
3.3 In the groups being compared (exposed vs non-exposed; with intervention vs without; cases vs controls), are the participants comparable, or do researchers take into account (control for) the difference between these groups?	Can’t tell	Did not report whether groups were comparable, no description of group characteristics.
3.4 Are there complete outcome data (80% or above), and, when applicable, an acceptable response rate (60% or above), or an acceptable follow-up rate for cohort studies (depending on the duration of follow-up)?	Yes	Delivery outcomes, maternal morbidity and fetal outcomes reported for both groups. All included are included in the analysis.
Overall quality score	*** 75%	
**Lelaidier, 1994**<br>**(Prospective double blind placebo controlled trial)**		
Are there clear qualitative and quantitative research questions (or objectives), or a clear mixed methods question (or objective)?	Yes	To evaluate the efficacy and tolerance of mifepristone in women undergoing induction of labor at term after previous cesarean section.
Do the collected data allow address the research question (objective)? E.g. consider whether the follow-up period is long enough for the outcome to occur (for longitudinal studies or study components).	Yes	Reported delivery, obstetric and neonatal outcomes.
2.1 Is there a clear description of the randomization (or an appropriate sequence generation)?	Yes	Double blind procedure, random permuted blocks of four.

2.2 Is there a clear description of the allocation concealment (or blinding when applicable)?	Yes	Supplied by pharmacy, external appearance of the tablets was similar.
2.3 Are there complete outcome data (80% or above)?	Yes	All outcomes appear reported.
2.4 Is there low withdrawal/drop-out (below 20%)?	Yes	Appears to have no withdrawals/drop-outs.
Overall quality score	**** 100%	
**Manish, 2016**<br>**(RCT)**		
Are there clear qualitative and quantitative research questions (or objectives), or a clear mixed methods question (or objective)?	Yes	To compare induction using Foley balloon inflated with 80mL vs. 30mL on vaginal delivery, and maternal and neonatal complications.
Do the collected data allow address the research question (objective)? E.g. consider whether the follow-up period is long enough for the outcome to occur (for longitudinal studies or study components).	Yes	Reported delivery, obstetric and neonatal outcomes.
2.1 Is there a clear description of the randomization (or an appropriate sequence generation)?	Yes	Random allocation sequence, permuted block randomization using SAS.
2.2 Is there a clear description of the allocation concealment (or blinding when applicable)?	Yes	Serially numbered, opaque, sealed envelopes.
2.3 Are there complete outcome data (80% or above)?	Yes	0 lost to follow-up, all outcomes appear reported.
2.4 Is there low withdrawal/drop-out (below 20%)?	Yes	0 lost to follow-up, appears no withdrawals.
Overall quality score	**** 100%	
**Ogbonmwan, 2010**<br>**(Retrospective cohort)**		
Are there clear qualitative and quantitative research questions (or objectives), or a clear mixed methods question (or objective)?	Yes	To compare outcomes of vaginal birth after primary cesarean with and without induction using prostaglandin and/or syntocinon augmentation.
Do the collected data allow address the research question (objective)? E.g. consider whether the follow-up period is long enough for the outcome to occur (for longitudinal studies or study components).	Yes	Reported delivery, obstetric and neonatal outcomes.
3.1 Are participants (organizations) recruited in a way that minimizes selection bias?	Yes	Review of obstetric records of all women at one hospital over 6-year period.
3.2 Are measurements appropriate (clear origin, or validity known, or standard instrument; and absence of contamination between groups when appropriate) regarding the exposure/intervention and outcomes?	Can't tell	Both groups, membrane sweep vs. spontaneous labor, also received multiple procedures for augmentation and analgesia, unclear if differences were significant.
3.3 In the groups being compared (exposed vs non-exposed; with intervention vs without; cases vs controls), are the participants comparable, or do researchers take into account (control for) the difference between these groups?	Can't tell	Did not report whether groups were comparable; Table "Maternal biodata" provides baseline demographics.
3.4 Are there complete outcome data (80% or above), and, when applicable, an acceptable response rate (60% or above), or an acceptable follow-up rate for cohort studies (depending on the duration of follow-up)?	Yes	Retrospective review of records, included all that fit inclusion criteria, reported delivery and maternal outcomes.
Overall quality score	** 50%	

**Palatnik, 2015**<br>**(Retrospective cohort)**		
Are there clear qualitative and quantitative research questions (or objectives), or a clear mixed methods question (or objective)?	Yes	To compare obstetric outcomes between women undergoing induction of labor and those undergoing expectant management >=39 weeks of gestation.
Do the collected data allow address the research question (objective)? E.g. consider whether the follow-up period is long enough for the outcome to occur (for longitudinal studies or study components).	Yes	Reported delivery, obstetric and neonatal outcomes.
3.1 Are participants (organizations) recruited in a way that minimizes selection bias?	Yes	Nation-wide registry from previous observational study.
3.2 Are measurements appropriate (clear origin, or validity known, or standard instrument; and absence of contamination between groups when appropriate) regarding the exposure/intervention and outcomes?	Yes	Rate of VBAC, no co-interventions reported in either groups.
3.3 In the groups being compared (exposed vs non-exposed; with intervention vs without; cases vs controls), are the participants comparable, or do researchers take into account (control for) the difference between these groups?	Can't tell	Reported characteristics of women for all comparison groups, indicating differences in age, race and obstetric history. No mention of controlling for any of these variables.
3.4 Are there complete outcome data (80% or above), and, when applicable, an acceptable response rate (60% or above), or an acceptable follow-up rate for cohort studies (depending on the duration of follow-up)?	Yes	All records used from registry were included. Reported delivery and maternal outcomes.
Overall quality score	*** 75%	
**Ramya, 2015**<br>**(RCT)**		
Are there clear qualitative and quantitative research questions (or objectives), or a clear mixed methods question (or objective)?	Yes	To investigate the effects of serial membrane sweeping on the onset of labor in women with previous LSCS who wished to undergo VBAC.
Do the collected data allow address the research question (objective)? E.g. consider whether the follow-up period is long enough for the outcome to occur (for longitudinal studies or study components).	Yes	Reported delivery, obstetric and neonatal outcomes.
2.1 Is there a clear description of the randomization (or an appropriate sequence generation)?	No	Randomization sequence generation NR.
2.2 Is there a clear description of the allocation concealment (or blinding when applicable)?	Yes	Sequential opening of numbered, sealed, opaque envelopes.
2.3 Are there complete outcome data (80% or above)?	Yes	All outcomes appear reported.
2.4 Is there low withdrawal/drop-out (below 20%)?	Yes	Appears to have no withdrawals/drop-outs.
Overall quality score	*** 75%	
**Rayburn, 1999**<br>**(RCT, multicenter)**		
Are there clear qualitative and quantitative research questions (or objectives), or a clear mixed methods question (or objective)?	Yes	To compare the clinical effectiveness of PGE2 gel at 39-41 weeks of gestation with that of expectant management for women who agreed to trials of labor after single cesareans.

Do the collected data allow address the research question (objective)? E.g. consider whether the follow-up period is long enough for the outcome to occur (for longitudinal studies or study components).	Yes	Reported delivery, obstetric and neonatal outcomes.
2.1 Is there a clear description of the randomization (or an appropriate sequence generation)?	Yes	Randomization generated by pharmaceutical company computer.
2.2 Is there a clear description of the allocation concealment (or blinding when applicable)?	Yes	Investigators masked to the assignments.
2.3 Are there complete outcome data (80% or above)?	Yes	All outcomes appear reported; all were monitored throughout study.
2.4 Is there low withdrawal/drop-out (below 20%)?	Yes	Mentions subject who dropped out after protocol treatment; performed intention to treat analysis, all followed up.
Overall quality score	**** 100%	
**Sakala, 1990a (Retrospective cohort)**		
Are there clear qualitative and quantitative research questions (or objectives), or a clear mixed methods question (or objective)?	Yes	To investigate the effects of oxytocin on chance of successful trial of labor, adverse effects and factors associated with failed trial of labor when oxytocin is used.
Do the collected data allow address the research question (objective)? E.g. consider whether the follow-up period is long enough for the outcome to occur (for longitudinal studies or study components).	Yes	Reported delivery, obstetric, maternal and neonatal outcomes.
3.1 Are participants (organizations) recruited in a way that minimizes selection bias?	Yes	Used database information, all women from one hospital with at least one previous cesarean delivery.
3.2 Are measurements appropriate (clear origin, or validity known, or standard instrument; and absence of contamination between groups when appropriate) regarding the exposure/intervention and outcomes?	Yes	No co-interventions reported for either group. Measuring trial of labor, VBAC rate and other measurements.
3.3 In the groups being compared (exposed vs non-exposed; with intervention vs without; cases vs controls), are the participants comparable, or do researchers take into account (control for) the difference between these groups?	Can't tell	Compared demographics between vaginal delivery & CS but not between oxytocin and no oxytocin.
3.4 Are there complete outcome data (80% or above), and, when applicable, an acceptable response rate (60% or above), or an acceptable follow-up rate for cohort studies (depending on the duration of follow-up)?	Yes	Appears no loss of follow-up due to retrospective nature, reported delivery, obstetric and maternal outcomes.
Overall quality score	*** 75%	
**Sakala, 1990b (Retrospective cohort)**		
Are there clear qualitative and quantitative research questions (or objectives), or a clear mixed methods question (or objective)?	Yes	To investigate the effects of epidural analgesia on chance of successful trial of labor, adverse effects, and factors associated with failed trial of labor when analgesia is used.
Do the collected data allow address the research question (objective)? E.g. consider whether the follow-up period is long enough for the outcome to	Yes	Reported delivery, obstetric, maternal and neonatal outcomes.

occur (for longitudinal studies or study components).		
3.1 Are participants (organizations) recruited in a way that minimizes selection bias?	Yes	All women from one hospital using data from perinatal database.
3.2 Are measurements appropriate (clear origin, or validity known, or standard instrument; and absence of contamination between groups when appropriate) regarding the exposure/intervention and outcomes?	No	Both groups (analgesia vs. no analgesia) also received oxytocin for augmentation (23% vs. 15%) and for induction (40% vs. 7%), no epidural analgesia group also received narcotic-sedative combination (69%); analgesia group did not appear to have received this as well.
3.3 In the groups being compared (exposed vs non-exposed; with intervention vs without; cases vs controls), are the participants comparable, or do researchers take into account (control for) the difference between these groups?	Can't tell	Compared demographics between epidural and no epidural – difference in cervical dilation on admission (less for former). Did not report controlling for differences.
3.4 Are there complete outcome data (80% or above), and, when applicable, an acceptable response rate (60% or above), or an acceptable follow-up rate for cohort studies (depending on the duration of follow-up)?	Yes	Appears no loss of follow-up due to retrospective nature, reported delivery, obstetric, maternal outcomes.
Overall quality score	** 50%	
**Shah, 2017 (Retrospective cohort)**		
Are there clear qualitative and quantitative research questions (or objectives), or a clear mixed methods question (or objective)?	Yes	To compare induction of labor methods in patients attempting a trial of labor after cesarean with an unfavorable cervix.
Do the collected data allow address the research question (objective)? E.g. consider whether the follow-up period is long enough for the outcome to occur (for longitudinal studies or study components).	Yes	Reported delivery, obstetric, maternal and neonatal outcomes.
3.1 Are participants (organizations) recruited in a way that minimizes selection bias?	Yes	Chart review, all women from one hospital using labor & delivery chart data of women with at least one prior cesarean delivery.
3.2 Are measurements appropriate (clear origin, or validity known, or standard instrument; and absence of contamination between groups when appropriate) regarding the exposure/intervention and outcomes?	Can't tell	Both groups (oxytocin vs. cook balloon) also received oxytocin (all women per group, p=0.24) and epidural anesthesia (92% vs 97%, p=0.24).
3.3 In the groups being compared (exposed vs non-exposed; with intervention vs without; cases vs controls), are the participants comparable, or do researchers take into account (control for) the difference between these groups?	Can't tell	Make note of differences in comparison groups, but do not account for it in analysis.
3.4 Are there complete outcome data (80% or above), and, when applicable, an acceptable response rate (60% or above), or an acceptable follow-up rate for cohort studies (depending on the duration of follow-up)?	Yes	Reported delivery, maternal, obstetric outcomes, all that fit inclusion criteria were included.
Overall quality score	** 50%	
**Shatz, 2013 (Retrospective cohort)**		

Are there clear qualitative and quantitative research questions (or objectives), or a clear mixed methods question (or objective)?	Yes	To determine the success rate of induction of labor in women with a prior cesarean section, and compare perinatal outcomes among women who had an induction of labor, spontaneous onset of labor or elective repeat cesarean section.
Do the collected data allow address the research question (objective)? E.g. consider whether the follow-up period is long enough for the outcome to occur (for longitudinal studies or study components).	Yes	Reported delivery, obstetric, maternal and neonatal outcomes.
3.1 Are participants (organizations) recruited in a way that minimizes selection bias?	Yes	Used medical center records, all women from one medical center using delivery records with a prior cesarean section.
3.2 Are measurements appropriate (clear origin, or validity known, or standard instrument; and absence of contamination between groups when appropriate) regarding the exposure/intervention and outcomes?	Yes	No co-interventions reported for either group; measured VBAC rate and delivery outcomes.
3.3 In the groups being compared (exposed vs non-exposed; with intervention vs without; cases vs controls), are the participants comparable, or do researchers take into account (control for) the difference between these groups?	Can't tell	Baseline characteristics show pretty similar, but no control for differences.
3.4 Are there complete outcome data (80% or above), and, when applicable, an acceptable response rate (60% or above), or an acceptable follow-up rate for cohort studies (depending on the duration of follow-up)?	Yes	Included all reports of those that fit inclusion criteria, reported delivery, maternal and obstetric outcomes.
Overall quality score	*** 75%	
**Sims, 2001**<br>**(Retrospective cohort)**		
Are there clear qualitative and quantitative research questions (or objectives), or a clear mixed methods question (or objective)?	Yes	To determine the impact of labor induction on both success and safety of trial of labor in candidates for vaginal birth after cesarean.
Do the collected data allow address the research question (objective)? E.g. consider whether the follow-up period is long enough for the outcome to occur (for longitudinal studies or study components).	Yes	Reported delivery, perinatal data from perinatal database.
3.1 Are participants (organizations) recruited in a way that minimizes selection bias?	Yes	Consecutive deliveries of women with previous cesarean delivery. Used network registry.
3.2 Are measurements appropriate (clear origin, or validity known, or standard instrument; and absence of contamination between groups when appropriate) regarding the exposure/intervention and outcomes?	No	Three possible interventions in "induction" group, plus co-intervention of oxytocin augmentation with misoprostol & dinoprostone; spontaneous labor group also received oxytocin for augmentation (number NR).
3.3 In the groups being compared (exposed vs non-exposed; with intervention vs without; cases vs controls), are the participants comparable, or do researchers take into account (control for) the difference between these groups?	No	Demographic data collected but not reported.

3.4 Are there complete outcome data (80% or above), and, when applicable, an acceptable response rate (60% or above), or an acceptable follow-up rate for cohort studies (depending on the duration of follow-up)?	Yes	From registry, seems to include all outcome data.
Overall quality score	** 50%	
**Taylor, 1993 (RCT)**		
Are there clear qualitative and quantitative research questions (or objectives), or a clear mixed methods question (or objective)?	Yes	To compare amniotomy and intravenous oxytocin infusion with vaginal prostaglandins followed by amniotomy to induce labor.
Do the collected data allow address the research question (objective)? E.g. consider whether the follow-up period is long enough for the outcome to occur (for longitudinal studies or study components).	Yes	Reported delivery outcomes, including neonatal Apgar scores and birthweight.
2.1 Is there a clear description of the randomization (or an appropriate sequence generation)?	Can’t tell	Used a predetermined code.
2.2 Is there a clear description of the allocation concealment (or blinding when applicable)?	Yes	Sealed envelopes, can assume opaque.
2.3 Are there complete outcome data (80% or above)?	Yes	All outcome data appear reported.
2.4 Is there low withdrawal/drop-out (below 20%)?	Yes	Appears to have no withdrawals/drop-outs.
Overall quality score	*** 75%	
**Tussupkaliyer, 2016 (Prospective cohort)**		
Are there clear qualitative and quantitative research questions (or objectives), or a clear mixed methods question (or objective)?	Yes	To assess the effectiveness and success rate of labor induction after previous CS.
Do the collected data allow address the research question (objective)? E.g. consider whether the follow-up period is long enough for the outcome to occur (for longitudinal studies or study components).	Yes	Reported delivery outcomes.
3.1 Are participants (organizations) recruited in a way that minimizes selection bias?	Can’t tell	No explanation of recruitment or selection process.
3.2 Are measurements appropriate (clear origin, or validity known, or standard instrument; and absence of contamination between groups when appropriate) regarding the exposure/intervention and outcomes?	No	Data distinguishes misoprostol vs. amniotomy vs. oxytocin vs. spontaneous labor. However, amniotomy and oxytocin groups are co-interventions that occurred serially.
3.3 In the groups being compared (exposed vs non-exposed; with intervention vs without; cases vs controls), are the participants comparable, or do researchers take into account (control for) the difference between these groups?	Yes	Groups were comparable in age/body weight/extragenital pathologies.
3.4 Are there complete outcome data (80% or above), and, when applicable, an acceptable response rate (60% or above), or an acceptable follow-up rate for cohort studies (depending on the duration of follow-up)?	Yes	All women in the cohorts are included in analysis. Patients followed to delivery.
Overall quality score	** 50%	
**Yogev, 2004 (Retrospective cohort)**		

Are there clear qualitative and quantitative research questions (or objectives), or a clear mixed methods question (or objective)?	Yes	To determine pregnancy outcome in women with one previous cesarean delivery in whom labor was induced in comparison with those who entered labor spontaneously.
Do the collected data allow address the research question (objective)? E.g. consider whether the follow-up period is long enough for the outcome to occur (for longitudinal studies or study components).	Yes	Reported delivery outcomes.
3.1 Are participants (organizations) recruited in a way that minimizes selection bias?	Yes	Consecutive women from one center with one previous cesarean section.
3.2 Are measurements appropriate (clear origin, or validity known, or standard instrument; and absence of contamination between groups when appropriate) regarding the exposure/intervention and outcomes?	Can't tell	Both PGE2 and spontaneous labor groups had same co-interventions (oxytocin or artificial rupture of membranes for labor augmentation). Oxytocin = 25% vs. 26% (p=0.75). Artificial rupture of membrane rates NR.
3.3 In the groups being compared (exposed vs non-exposed; with intervention vs without; cases vs controls), are the participants comparable, or do researchers take into account (control for) the difference between these groups?	Yes	There was no between group differences in maternal age, gravidity or age.
3.4 Are there complete outcome data (80% or above), and, when applicable, an acceptable response rate (60% or above), or an acceptable follow-up rate for cohort studies (depending on the duration of follow-up)?	Yes	Used retrospective study design, appears to have no loss to follow-up, all those included were included in the analysis.
Overall quality score	*** 75%	

* Assessed using the Mixed Methods Appraisal Tool, Version 2011

LSTCS: lower section transverse cesarean section; LSCS: lower section cesarean section; NR: not reported; PGE1/PGE2: prostaglandin E1/E2; SAS: statistical analysis system; VBAC: vaginal birth after cesarean
